# Unsupervised Anomaly Detection in Medical Imaging: A Survey of Methods, Challenges, and Future Directions

**DOI:** 10.3390/bioengineering13050547

**Published:** 2026-05-11

**Authors:** Boyang Liu, Guangli Li, Yuxing Zou, Shiying Zeng, Jingqin Lv, Renzhong Wu, Hongbin Zhang

**Affiliations:** School of Information and Software Engineering, East China Jiaotong University, Nanchang 330013, China; 2023068081200022@ecjtu.edu.cn (B.L.); 2023068081200023@ecjtu.edu.cn (Y.Z.); zengshiying@jgsu.edu.cn (S.Z.); jingqinlv@ecjtu.edu.cn (J.L.); wurenzhong@csu.edu.cn (R.W.); zhanghongbin@whu.edu.cn (H.Z.)

**Keywords:** medical imaging, unsupervised learning, anomaly detection, deep learning, computer-aided diagnosis

## Abstract

Unsupervised anomaly detection in medical imaging aims to automatically identify potential lesions that deviate from normal patterns in multimodal medical images without requiring annotations of abnormal samples, and is of great clinical value for early disease screening, unknown anomaly discovery, and label-scarce or open-set detection scenarios. Compared with industrial anomaly detection, medical images are characterized by complex anatomical structures, high semantic complexity of abnormalities, substantial inter-individual variability, and high annotation costs, which make the modeling and evaluation of related methods more challenging. This review systematically surveys unsupervised anomaly detection methods for medical imaging. By integrating task definitions, technological evolution, and clinical application needs, we comprehensively analyze 149 representative studies and 16 commonly used datasets collected from major academic databases. First, according to their core modeling paradigms, existing mainstream methods are categorized into four groups: image reconstruction-based methods, feature embedding-based methods, self-supervised learning-based methods, and foundation model-based methods. The technical characteristics, applicable scenarios, and inherent limitations of each category are then systematically discussed. Furthermore, from the perspectives of medical image structural properties and clinical application requirements, we summarize the key challenges currently faced by medical image unsupervised anomaly detection, including abnormal semantic modeling, the reliability of anomaly quantification, cross-center generalization capability, and evaluation protocols. Finally, future research directions, such as multi-task modeling, medical prior-guided learning, and multimodal fusion, are discussed in depth.

## 1. Introduction

Medical imaging serves as a fundamental basis for modern medical diagnosis, treatment planning, and disease management. With the widespread use of various imaging modalities such as computed tomography (CT), magnetic resonance imaging (MRI), X-ray, endoscopy, and histopathological slides, the volume of medical imaging data has increased dramatically. However, image interpretation still largely relies on the experience and subjective judgment of clinicians, which is not only time-consuming and labor-intensive but also susceptible to fatigue and inter-observer variability. Therefore, automatically identifying potential abnormalities from massive medical imaging data using artificial intelligence (AI) techniques has become an important research direction in intelligent medical image analysis [1].

In medical image analysis, anomaly detection (AD) aims to identify abnormal samples or regions that deviate from normal patterns [2]. Compared with traditional medical image classification or lesion segmentation tasks, medical imaging anomaly detection is more consistent with the actual clinical diagnostic workflow. When analyzing medical images, clinicians usually do not directly determine the disease category. Instead, they first identify suspicious abnormal manifestations and subsequently infer possible pathological conditions based on their morphology, intensity, or structural characteristics. Moreover, the types of abnormalities encountered in real-world clinical cases are often open-ended and uncertain. Consequently, anomaly detection methods can mimic this clinical reasoning process of “detection before diagnosis,” demonstrating stronger generalization capability for complex, unknown, or rare pathological patterns. In tasks such as early disease screening (e.g., brain tumors, pulmonary nodules, and retinal disorders), intraoperative navigation, lesion monitoring, and imaging quality control, anomaly detection has shown significant clinical value.

However, medical imaging data typically exhibit high dimensionality, high noise levels, and strong structural correlations. In addition, physiological variations across individuals lead to substantial diversity in the distribution of normal samples themselves [3]. Meanwhile, abnormal samples in real clinical datasets are usually scarce and highly imbalanced. Certain rare diseases or early-stage lesions account for only a very small proportion of the data. The annotation of such abnormalities often requires expert clinicians to perform fine-grained delineation or grading, which is costly and subject to subjective variability. Furthermore, the types of abnormalities encountered in clinical practice continuously evolve, and newly emerging pathological patterns may not be included in existing category systems. These factors collectively make it difficult for traditional supervised learning methods, which rely heavily on large numbers of labeled abnormal samples, to be reliably applied in medical scenarios.

Against this background, increasing attention has been directed toward medical imaging unsupervised anomaly detection (MUAD). Such methods follow the core idea of learning only the distribution of normal samples. By modeling the statistical characteristics or semantic representations of normal images (i.e., images without significant abnormal structures or pathological signs), they detect anomalous samples that deviate from the learned normal distribution during inference [4]. Unsupervised anomaly detection first achieved remarkable progress in the field of industrial visual inspection and was subsequently gradually transferred to medical imaging applications. Since normal medical images can be continuously collected through routine examinations and screenings, whereas abnormal samples are relatively scarce and highly diverse in appearance, MUAD provides a more scalable technical paradigm for addressing the challenges of limited abnormal data and open-set anomalies in medical imaging. In essence, MUAD aims to detect and localize potential abnormalities without relying on large amounts of annotated anomalous data, making it particularly suitable for real-world clinical scenarios where abnormal annotations are scarce or incomplete. Such capability is especially important in applications such as early disease screening, rare disease detection, and imaging-based monitoring, where unknown or subtle abnormalities need to be identified in a data-driven manner. Accordingly, MUAD has demonstrated promising potential in real-world clinical applications.

**Figure 1:**
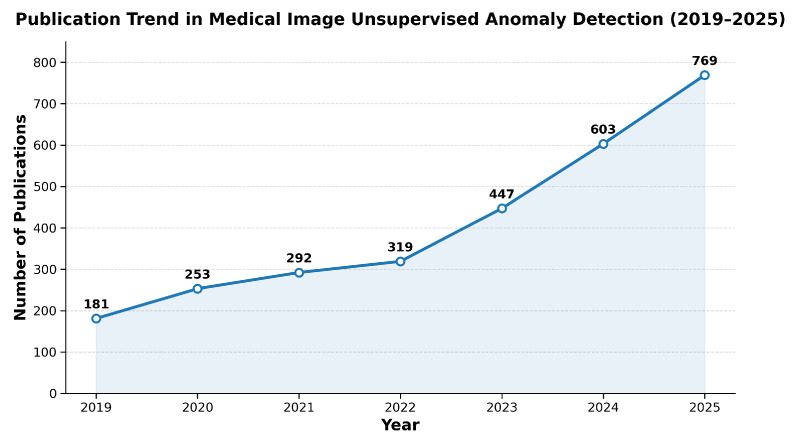
Annual number of publications in medical image unsupervised anomaly detection from 2019 to 2025. Data were collected from the Semantic Scholar database based on keyword retrieval.

Existing survey studies [5,6] mainly focus on industrial image anomaly detection (industrial imaging unsupervised anomaly detection, IUAD) or specific benchmark datasets (such as MVTec AD [7] and VisA [8]). In contrast, systematic reviews dedicated to MUAD remain relatively limited. Although a few surveys on medical anomaly detection have been reported [2,9], they were either published relatively early and therefore cannot cover the rapidly emerging methods in recent years, or primarily concentrate on a single technical paradigm (e.g., image reconstruction-based approaches), lacking a comprehensive summary of multiple mainstream methodologies. Meanwhile, with the rapid development of deep learning techniques, MUAD has gradually become an important research topic in medical artificial intelligence. As illustrated in Figure 1, the number of related publications from 2019 to 2025 has steadily increased, reflecting growing research interest in this field. However, existing studies often discuss methods in an isolated manner, without providing a unified perspective to understand how different modeling paradigms address the core challenges of MUAD. Therefore, a structured and mechanism-oriented survey is needed to systematically organize existing methods and clarify their relationships, strengths, and limitations. Furthermore, medical imaging anomaly detection differs significantly from industrial visual inspection tasks in several aspects. Medical abnormalities often manifest as subtle structural variations with complex morphology and ambiguous semantics, and they are influenced by individual physiological differences, variations in imaging conditions, and clinical interpretability requirements. These characteristics pose greater challenges for feature modeling and performance evaluation. Therefore, it is necessary to systematically review MUAD methods from the perspective of medical imaging applications; clarify their technical characteristics, applicable scenarios, and development trends; and provide guidance for method design, clinical deployment, and future research directions.

**Figure 2:**
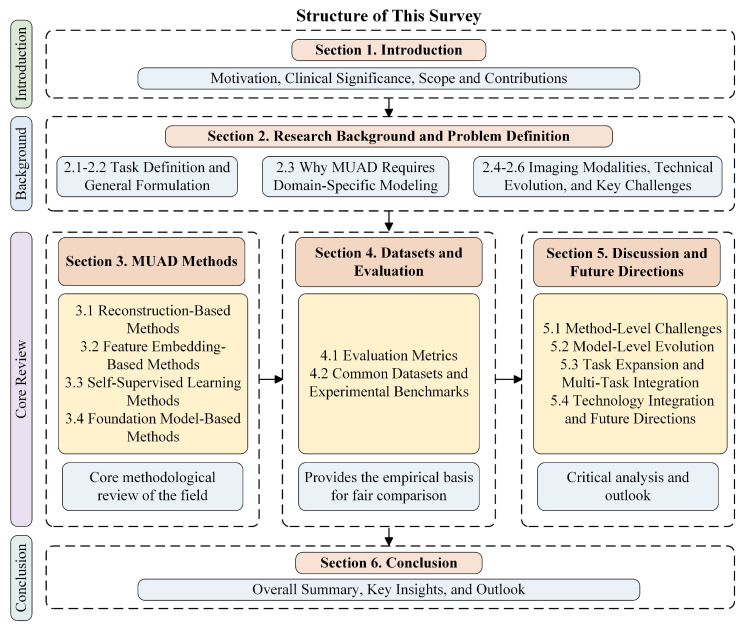
Structure of this survey. The paper is organized from the research background and problem definition of medical imaging unsupervised anomaly detection (MUAD), to a systematic review of methodological paradigms, followed by benchmark datasets and evaluation, and finally a discussion of current challenges and future research directions.

Motivated by these considerations, this paper systematically reviews 149 representative studies and 16 commonly used public datasets. To provide a clear and coherent understanding of the field, this survey follows the general organization commonly adopted in recent survey studies [2,3,9]. We first define the MUAD problem and summarize its unique challenges. We then systematically review existing methods according to their underlying modeling mechanisms. Next, we analyze how different categories of methods address the core difficulties of MUAD and discuss their strengths and limitations. Finally, we summarize open problems and future research directions. Figure 2 illustrates the overall structure of this survey. The main contributions of this survey are summarized as follows:

- We clarify the problem setting, clinical significance, and representative application scenarios of medical imaging unsupervised anomaly detection. Building on this, we compare MUAD with industrial image anomaly detection from a cross-domain perspective to highlight the distinctive challenges of medical imaging, including complex anomaly semantics, substantial domain shifts, and limited annotation resources.
- We present a systematic taxonomy of existing MUAD approaches, categorizing them into four major paradigms: image reconstruction, feature embedding, self-supervised learning, and foundation-model-based methods. In addition, we summarize representative models, widely used benchmark datasets, and evaluation metrics, providing a comprehensive overview of the MUAD research landscape.
- We analyze key challenges in current MUAD research, including reconstruction bias, domain discrepancies, reliability of anomaly scoring, and inconsistencies in evaluation protocols. Furthermore, we discuss several promising future research directions, such as cross-modal fusion, knowledge-guided learning, and the development of medical foundation models.

## 2. Research Background and Problem Definition

### 2.1. Task Definition

Medical imaging anomaly detection is an important research direction in medical artificial intelligence. Its objective is to automatically identify regions that deviate from normal anatomical or physiological patterns in various medical imaging modalities, such as CT, MRI, X-ray, and ultrasound, thereby enabling image-level anomaly detection or pixel-level lesion localization [10]. These abnormal deviations usually correspond to potential pathological conditions or functional abnormalities of tissues, including tumors, ischemia, hemorrhage, inflammation, or other pathological alterations, which serve as crucial evidence for clinical decision-making. Therefore, medical imaging anomaly detection is considered one of the key tasks in computer-aided diagnosis (CAD).

In practical applications, the primary goal of medical imaging anomaly detection is to identify various potential disease-related abnormalities from medical images. However, clinical datasets generally exhibit characteristics such as scarcity of abnormal samples, incomplete annotations, and severe class imbalance, making traditional supervised methods that rely on large amounts of labeled data difficult to apply effectively [3]. In this context, medical imaging unsupervised anomaly detection (MUAD) has emerged as a promising paradigm. The fundamental idea of MUAD is to learn the statistical distribution or semantic representations of normal tissues using only normal samples during training, and to identify potential lesions during inference through reconstruction errors, probabilistic deviations, or abnormal patterns in feature space [11].

Furthermore, medical anomalies often exhibit open-set characteristics and complex data distributions, which makes conventional modeling approaches based on fixed category assumptions insufficient to fully capture real clinical scenarios. By modeling the distribution of normal data to detect deviations, MUAD not only extends the research paradigm of open-set anomaly detection but also better aligns with practical clinical needs, such as early disease screening and the discovery of previously unseen abnormalities. Consequently, MUAD has significant research value and promising clinical application potential.

**Figure 3:**
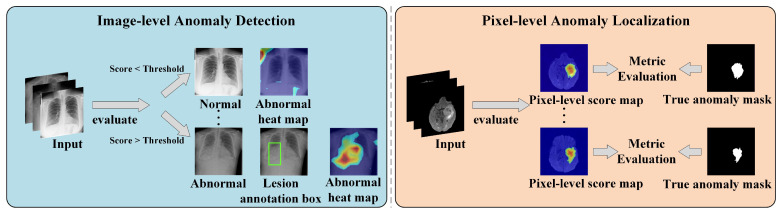
Illustration of image-level anomaly detection and pixel-level anomaly localization. Image-level anomaly detection classifies images according to a global anomaly score with a predefined threshold and highlights potential abnormal regions using heatmaps. Pixel-level anomaly localization generates a pixel-wise anomaly score map (e.g., anomaly saliency or probability map) to delineate abnormal regions, which is evaluated against the ground-truth mask using metrics such as Dice, IoU, and AUPRO. Green boxes indicate the ground-truth anomaly bounding boxes provided in the dataset. Warmer colors in the heatmaps represent higher anomaly scores.

As illustrated in Figure 3, according to the detection granularity, MUAD tasks can generally be divided into two categories.

1. **Image-level unsupervised anomaly detection (Image-level UAD):** This task aims to determine whether an entire image deviates from the normal distribution. When trained only on normal samples, the model outputs a global anomaly score for each input image, and a predefined threshold is used to classify the image as normal or abnormal. This task is commonly applied in practical scenarios such as disease screening, medical image quality control, and diagnostic assistance. From a methodological perspective, image-level anomaly detection is closely related to paradigms such as one-class detection and out-of-distribution detection, rather than traditional medical image classification.
2. **Pixel-level unsupervised anomaly detection (Pixel-level UAD):** This task aims to automatically generate anomaly saliency maps or probability maps without relying on pixel-level annotations, thereby locating potential lesion regions and analyzing local structural deviations. Compared with image-level detection, this task not only determines whether anomalies exist but also emphasizes the spatial distribution of abnormal regions. It is commonly used for lesion localization, boundary analysis, and lesion volume quantification, and can essentially be regarded as a form of anomaly saliency detection.

Although image-level and pixel-level tasks differ in their output formats, they are not entirely independent in practical method frameworks. In general, these two tasks differ mainly in terms of granularity and evaluation protocols: image-level tasks focus on the accuracy of detecting abnormal images, whereas pixel-level tasks emphasize the ability to localize and segment anomalous regions. Nevertheless, in most MUAD approaches, models learn the distribution of normal samples and generate both image-level anomaly scores and pixel-level anomaly maps within a unified framework using different anomaly measurement strategies. Therefore, these two tasks can be regarded as different perspectives within the same unsupervised anomaly detection framework, both built upon the learning of normal patterns and jointly forming the primary task paradigms in current MUAD research.

### 2.2. General Formulation of MUAD

To provide a unified modeling perspective for the diverse MUAD methods reviewed in this survey, we briefly formulate the general learning objective and inference process of medical imaging unsupervised anomaly detection. In MUAD, the training set usually contains only normal samples, which can be denoted as

(1) $$D_{tr}={\left\{x_{i}\right\}}_{i=1}^{N},\;x_{i}∼p_{n}\left(x\right),$$

where $p_{n}\left(x\right)$ denotes the distribution of normal medical images. The goal is to learn a model $f_{\theta }$ from normal data:

(2) $$\theta^{*}=\arg\min_{\theta }L\left(f_{\theta };D_{tr}\right),$$

where L(·) denotes the training objective, which may vary across different methodological paradigms.

During inference, a test image *x* is assigned an anomaly score

(3) $$s\left(x\right)=\phi \left(f_{\theta^{*}}\left(x\right)\right),$$

where ϕ(·) denotes the anomaly scoring function. For image-level anomaly detection, the prediction is obtained by thresholding the anomaly score:

(4) $$\hat{y}=\left\{\begin{array}{cc} 1, & s(x)>\tau ,\\ 0, & s(x)\leq \tau , \end{array}\right.$$

where $\hat{y}=1$ and $\hat{y}=0$ indicate abnormal and normal images, respectively. For pixel-level anomaly localization, the model further produces an anomaly map

(5) $$A\left(x\right)\in R^{H\times W},$$

where each spatial location reflects the degree of abnormality of the corresponding region. Under this formulation, most MUAD methods can be viewed within a common framework of normal pattern learning, anomaly scoring, and final image-level or pixel-level prediction.

**Figure 4:**
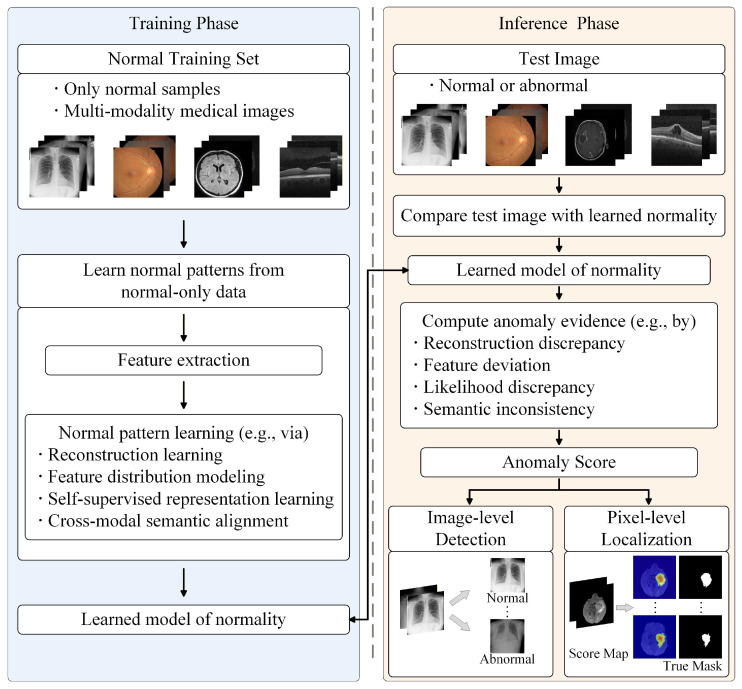
General pipeline of medical imaging unsupervised anomaly detection (MUAD), including the training phase of normal pattern learning and the inference phase of anomaly scoring and prediction.

The general formulation above can be further illustrated from the perspective of the overall MUAD pipeline. As shown in Figure 4, MUAD usually consists of two stages: in the training phase, the model learns normal patterns from normal-only medical images; in the inference phase, the test image is compared with the learned normality model to produce anomaly evidence and corresponding anomaly scores, which are then used for image-level detection or pixel-level localization.

### 2.3. Why Medical Anomaly Detection Requires Domain-Specific Modeling

Medical anomaly detection is closely related to the broader development of visual anomaly detection, yet its problem setting is substantially more complex. Early anomaly detection research was often explored in industrial inspection, where images are usually acquired under relatively controlled conditions and anomalies tend to present clearer visual patterns. Such settings provided an important basis for the development of unsupervised anomaly detection methods. However, the purpose of introducing these related domains here is not to shift the focus away from medical imaging, but to clarify the unique challenges and modeling demands of MUAD.

**Figure 5:**
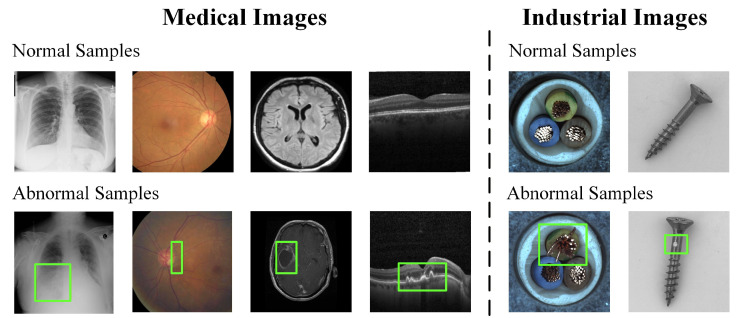
Comparison diagram of medical images and industrial images. The green box represents the abnormal area.

Although both MUAD and IUAD aim to identify abnormal patterns from images, and IUAD has formed a relatively mature methodological framework, the two domains differ fundamentally in anomaly characteristics and task requirements. As illustrated in Figure 5, industrial defects are often localized, visually explicit, and associated with relatively stable textures or structures. In contrast, medical abnormalities are usually embedded in complex anatomical contexts and may appear as subtle structural or semantic deviations with ambiguous boundaries, multi-scale variations, and strong dependence on imaging modality and individual physiology. These differences make it difficult to directly transfer IUAD methods to medical imaging scenarios. Beyond these visual differences, MUAD and IUAD also differ substantially in data properties and modeling priorities. As summarized in Table 1, medical images involve greater modality diversity, stronger inter-subject variability, scarcer abnormal samples, and much higher annotation costs due to the need for expert knowledge as well as privacy and ethical constraints. In addition, MUAD places greater emphasis on semantic structure modeling, clinical interpretability, and reliability, whereas IUAD more often focuses on texture representation, computational efficiency, and deployment stability.

Related anomaly detection studies have also been developed in remote sensing and video understanding [12,13,14]. Remote sensing anomaly detection often focuses on spectrally rare targets that deviate from the surrounding background, while video anomaly detection mainly concerns abnormal events in dynamic scenes and thus relies on spatiotemporal modeling. By comparison, MUAD is more strongly constrained by anatomical priors, modality heterogeneity, and the clinical semantics of abnormalities.

Overall, methods from industrial inspection, remote sensing, and video analysis provide useful inspiration for MUAD, such as robust representation learning, effective learning strategies, and recent foundation-model-based paradigms. Nevertheless, these methods cannot be directly applied to medical imaging without adaptation. Their transfer to MUAD requires explicit consideration of medical-specific challenges, including anatomical complexity, semantic ambiguity, modality diversity, and clinical interpretability.

### 2.4. Medical Imaging Modalities and Application Scenarios

Beyond differences in task objectives and data characteristics, medical imaging anomaly detection also faces more complex data source issues. Different medical imaging modalities exhibit significant variations in imaging mechanisms, structural representations, and noise characteristics, which directly influence the task focus and technical challenges of MUAD methods under different modalities.

For example, X-ray images mainly represent projection structures, CT and MRI emphasize three-dimensional anatomical consistency, histopathological and dermoscopic images present fine-grained textures, while endoscopy, ultrasound, and ophthalmic images are often significantly affected by illumination variations or imaging noise.

Therefore, understanding the imaging characteristics and abnormal manifestations across different modalities is crucial for the design and evaluation of medical imaging anomaly detection methods. Table 2 summarizes the major medical imaging modalities involved in anomaly detection research, along with their typical anomalies, imaging characteristics, modality-specific challenges, and commonly used datasets. This provides a unified background for subsequent methodological discussions.

In summary, MUAD has been explored across nearly all mainstream medical imaging modalities. From two-dimensional imaging such as X-ray, retinal fundus images, and endoscopic images, to three-dimensional modalities such as CT and MRI, and even gigapixel-scale histopathological whole slide images, unsupervised anomaly detection methods have demonstrated broad applicability across diverse imaging scenarios. A large body of research indicates that these methods show promising potential in tasks such as anomaly screening, early lesion discovery, and large-scale clinical data pre-screening. Their cross-modality applicability further highlights the importance of unsupervised anomaly detection in addressing key challenges in medical imaging, including data imbalance, rarity of abnormal samples, and limited annotation availability. In addition, from an application-oriented perspective, such cross-modality adaptability also suggests potential value for practical scenarios including suspicious case flagging, triage support, candidate lesion prompting, and image quality control [15]. For example, MUAD models with cross-scale adaptation ability may support suspicious case flagging by capturing both coarse structural abnormalities and subtle local lesions, while also providing candidate lesion prompts by highlighting anomalous regions across different spatial scales. However, robust deployment in real clinical workflows still requires further validation under heterogeneous real-world conditions.

**Table 2:** Summary of typical anomalies, imaging characteristics, challenges, datasets, and representative methods across different medical imaging modalities.

Modality	Region	Anomalies	Features	Challenges	Datasets	Methods
X-ray	Chest, lungs, bones, breast	Pulmonary nodules, pneumonia, pleural effusion, cardiomegaly, fractures, periosteal reactions, bone injuries, masses, calcifications, etc.	Two-dimensional projection imaging; diverse anomaly patterns; significant tissue overlap.	(1) Structural overlap caused by projection imaging may obscure lesions, especially behind bones. (2) Small abnormalities are difficult to detect. (3) Imaging posture variations lead to substantial differences even among normal images.	RSNA, VinDr-CXR [16], CheXpert [17], GRAZPEDWRI-DX [18]	SimSID [19], AMAE [20]
MRI	Brain	Brain tumors, multiple sclerosis lesions, demyelinating lesions, etc.	Multi-sequence imaging (e.g., T1/T2/FLAIR); high soft-tissue contrast; semantically apparent abnormalities.	(1) Significant inter-subject variability in brain structures leads to a complex normal distribution. (2) Minor patient motion or changes in imaging conditions may blur structural details.	Brain Tumor datasets, BraTS2021 [21]	StyleGAN [22], AutoDDPM [23], Pinaya et al. [24], Brainomaly [25]
CT	Lungs, kidneys	COVID-19 infection, pneumonia, pulmonary nodules, kidney tumors	Volumetric imaging with high resolution and relatively stable density differences.	(1) Large anatomical variability across individuals makes it difficult to model a unified normal pattern. (2) Some lesions, such as ground-glass nodules, have intensities close to those of normal tissues and often exhibit blurred boundaries.	LUNA16 [26], KiTS [27]	Kim et al. [28,29], TS-AC [30]
Retinal Fundus	Retina, optic disc	Glaucoma-related structural abnormalities, diabetic retinopathy	Prominent vascular textures and relatively clear geometric anatomical structures.	(1) Vascular structures vary considerably across individuals. (2) Illumination is often uneven and image contrast varies significantly.	LAG [31]	Zhang [32], Das et al. [33]
OCT	Layered retinal structures	Macular holes, retinal fluid accumulation, edema, retinal detachment	Pronounced layered structural changes with clear depth-wise characteristics.	(1) Image quality is sensitive to eye motion and scanning depth. (2) The normal layered retinal structure is complex, and minor deviations may be falsely recognized as anomalies.	OCT2017 [34]	Hetero-AE [35], ESC-DRKD [36]
Dermatoscopy	Skin surface, pigmented regions	Melanoma, benign nevus, keratosis, dermatofibroma	Appearance is mainly dominated by color and texture, with prominent variations in lesion boundaries and morphology.	(1) Lesion textures are highly diverse. (2) Illumination conditions and acquisition devices vary significantly.	ISIC2018 [37]	AE-FLOW [38], EA2D [39]
Ultrasound	Breast	Malignant masses	Strong speckle noise, blurred boundaries, and low contrast.	(1) Noise and artifacts are highly random. (2) Abnormal boundaries are often ambiguous, making precise localization difficult.	Breast Ultrasound Dataset [40]	ADFA [41], VisualAD [42]
Endoscopy	Gastrointestinal tract, esophagus, colorectum	Polyps, ulcers, mucosal hyperemia	Polyps vary greatly in size and exhibit complex surface structures.	(1) Flat polyps or subtle abnormalities are extremely difficult to perceive. (2) Illumination is unstable, and specular reflections, fluids, or metallic artifacts may interfere with analysis.	Kvasir [43], Kvasir-SEG [44], Hyper-Kvasir [45]	Huang et al. [46], PMSACL [47]
WSI	Tissue sections (e.g., breast, prostate)	Cancer cell clusters, abnormal mitosis, tissue architecture disorder	Extremely high resolution, multi-scale tissue organization, and fine-grained textures.	(1) The image size is extremely large, making it difficult to directly process the whole slide. (2) High variability among normal tissues makes normal distribution modeling challenging.	Camelyon16 [48]	GraphTox [49]

### 2.5. Technical Evolution

Early studies on MUAD mainly relied on traditional machine learning techniques, such as principal component analysis (PCA) [50], Gaussian mixture models, and sparse coding [51]. These approaches attempted to identify anomalies by describing the low-dimensional statistical characteristics of normal samples. While such methods can achieve reasonable performance in tasks with relatively regular structures, they often struggle to capture deeper semantic variations when dealing with the high dimensionality, complex morphology, and inter-subject variability inherent in medical imaging data. With the rapid development of deep learning, medical imaging anomaly detection methods have gradually evolved from shallow statistical modeling toward deep semantic representation learning. Early deep-learning-based approaches were primarily reconstruction-based methods built upon generative models, including Autoencoders (AE) [52], Variational Autoencoders (VAE) [53], Generative Adversarial Networks (GAN) [54], and Denoising Diffusion Probabilistic Models (DDPM) [10,55]. These models learn the generative mapping of normal samples and quantify anomalies using reconstruction errors between the input and reconstructed images. Subsequently, researchers realized that medical anomalies often involve anatomical structure variations and high-level semantic differences. Therefore, relying solely on reconstruction is insufficient to accurately characterize the distribution of normal medical images. This observation has led to the rapid development of feature embedding-based approaches, which leverage pretrained models to model normal patterns in deep feature spaces. Typical strategies include teacher–student frameworks [56,57], patch-level distribution modeling [58,59], memory bank mechanisms [60], and normalizing flow models [61,62]. By learning the distribution of normal samples in high-level feature spaces, these methods significantly improve the robustness and generalization capability of anomaly detection models. To further enhance semantic representation learning under the absence of labeled anomalies, self-supervised learning [63,64,65,66] and contrastive learning [67,68] approaches have attracted increasing attention. These methods construct auxiliary proxy tasks or feature-level contrastive constraints, enabling models to automatically learn semantic representations that are sensitive to anomalies without relying on manual annotations. More recently, with the rapid development of the foundation model paradigm, researchers have begun to introduce large-scale pretrained models, including vision–language models and large language models [69,70]. By jointly modeling images, textual descriptions, and clinical knowledge, these approaches aim to enhance semantic understanding, improve model interpretability, and ultimately increase the clinical applicability of anomaly detection systems.

**Figure 6:**
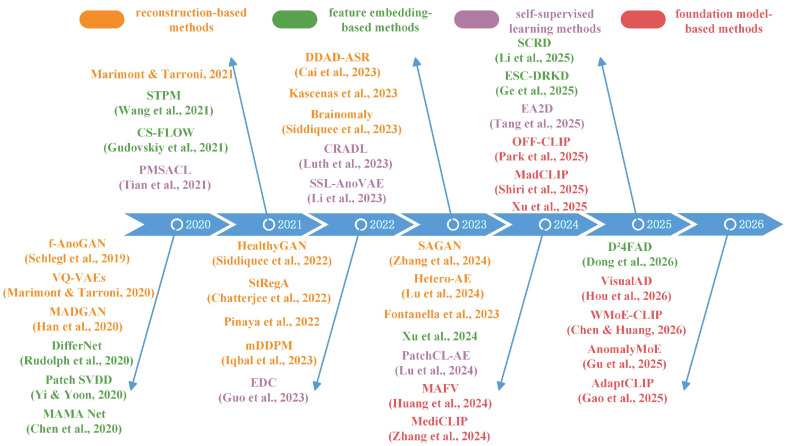
A schematic diagram illustrating the development trend of unsupervised anomaly detection methods for medical images from 2020 to 2026. Different colors represent reconstruction-based methods, feature embedding-based methods, self-supervised learning approaches, and foundation model-based approaches, respectively. The methods shown and their corresponding references are: f-AnoGAN [71], VQ-VAEs [72], MADGAN [73], DifferNet [74], Patch SVDD [75], MAMA Net [76], Marimont & Tarroni, 2021 [77], STPM [78], CS-FLOW [79], PMSACL [47], HealthyGAN [80], StRegA [81], Pinaya et al., 2022 [24], mDDPM [82], EDC [83], DDAD-ASR [84], Kascenas et al., 2023 [85], Brainomaly [25], CRADL [86], SSL-AnoVAE [87], SAGAN [88], Hetero-AE [35], Fontanella et al., 2023 [89], Xu et al., 2024 [90], PatchCL-AE [91], MAFV [92], MediCLIP [93], D^2^4FAD [94], VisualAD [42], WMoE-CLIP [95], AnomalyMoE [96], AdaptCLIP [97], SCRD [98], ESC-DRKD [36], EA2D [39], OFF-CLIP [99], MadCLIP [100], Xu et al., 2025 [101]. For works initially released as preprints, the earliest publicly available version year is used; minor discrepancies with formal publication dates may exist.

As shown in Figure 6, MUAD research has gradually evolved from early reconstruction-driven generative models toward approaches that model deep feature distributions, and has further integrated techniques such as self-supervised learning, diffusion models, and cross-modal foundation models. This evolution reflects a clear transition from low-level pixel modeling to high-level semantic understanding. Overall, MUAD has progressed from early “pixel reconstruction” paradigms to more comprehensive frameworks that integrate generative modeling, feature matching, and cross-modal semantic understanding. Correspondingly, the core objective of MUAD has gradually shifted from merely “detecting anomalies” toward “understanding anomalies”, providing a clearer technological trajectory for the development of future methods.

### 2.6. Key Challenges in MUAD Research

In recent years, although MUAD has made notable progress in method design and application scenarios, existing studies still face a number of critical challenges due to the complexity of medical data and the inherent constraints of the unsupervised learning paradigm.

1. **Complex medical image structures and significant intrinsic variability.** Medical images exhibit natural multi-scale structural hierarchies and strong tissue heterogeneity, covering morphological characteristics at the organ, tissue, and even cellular levels. Different scales often differ substantially in terms of spatial organization and texture patterns. As illustrated in Figure 5, chest X-ray images present global anatomical contours, brain MRI reflects complex brain region structures, and OCT images display highly layered tissue morphology. Therefore, even normal anatomical structures already exhibit highly complex hierarchical organization. In addition, substantial inter-subject variations exist in anatomical structures, physiological conditions, and imaging settings, resulting in highly dispersed distributions of normal samples and making it difficult to establish a unified and stable model of normality. Moreover, imaging devices, acquisition protocols, and imaging artifacts may introduce non-pathological variations that resemble lesion appearance, further interfering with anomaly discrimination. This issue is one of the core challenges that subsequent reconstruction-based and feature embedding-based methods attempt to address.
2. **Strong semantic properties and diverse manifestations of medical anomalies.** Abnormalities in medical images usually correspond to specific pathological types or disease states, and their identification is therefore closer to disease recognition than to simple visual outlier detection. As illustrated in Figure 5, many lesions do not manifest as obvious intensity or texture abnormalities; instead, they are reflected as structural deformations or abnormal tissue distributions, such as local compression caused by intracranial space-occupying lesions or disorganization of retinal layer structures. At the same time, the normal anatomical structures of different organs vary substantially, and anomalies themselves are highly diverse in scale, shape, and contrast. Consequently, unsupervised methods that rely only on low-level visual features or statistical deviations often fail to robustly characterize the essence of abnormalities, which also limits the semantic interpretability of anomaly detection results. How to move beyond low-level reconstruction toward high-level semantic modeling has therefore become an important direction in the subsequent evolution of MUAD methods.
3. **Inherent limitations of unsupervised anomaly detection methods.** Under the unsupervised setting for medical anomaly detection, most methods identify anomalies based on reconstruction errors, feature deviations, or similarity discrepancies. However, reconstruction-based methods are prone to partially reconstructing abnormal regions, which weakens anomaly contrast. Feature modeling methods, on the other hand, are easily affected by domain shifts and the limited suitability of pretrained models, resulting in unstable decision boundaries. In addition, self-supervised and multimodal methods still face limitations in pseudo-anomaly construction and semantic alignment. Meanwhile, features pretrained on natural images often have limited ability to represent low-contrast and weak-boundary medical structures, further reducing the reliability of anomaly measurement. These limitations have also driven the continued development of feature embedding-based, self-supervised, and foundation model-based methods.
4. **Scarce annotations and limitations of evaluation metrics.** Although unsupervised anomaly detection does not rely on abnormal annotations during training, reliable model evaluation still requires sufficiently accurate annotations as reference standards. However, high-quality annotations in medical imaging, especially pixel-level labels, are extremely scarce. In addition, some commonly used evaluation metrics may fail to objectively reflect clinical requirements when anomalous regions are sparse or highly localized. Under this circumstance, improving the quality of learned representations has become an important focus of self-supervised learning methods.
5. **Insufficient generalization and limited clinical interpretability.** Early MUAD methods were typically evaluated on a single imaging modality, whereas more recent studies have begun to explore multi-modality and cross-domain validation. However, achieving robust generalization across different medical imaging settings remains an open challenge. In practice, model performance may still degrade under cross-modality, cross-organ, multi-center, and cross-protocol scenarios, due to substantial differences in imaging mechanisms, anatomical structures, organ characteristics, acquisition devices, and population distributions. In addition, abnormal patterns in medical images are often highly heterogeneous and context-dependent, which further increases the difficulty of learning transferable representations of normality and abnormality. Meanwhile, model outputs are still commonly presented as anomaly scores or heatmaps, which are often difficult to align directly with pathological semantics and clinical diagnostic reasoning. This gap limits the practical clinical usability of current MUAD systems. These challenges have also motivated the gradual introduction of foundation models to improve cross-modal generalization and high-level semantic interpretability.

## 3. Medical Imaging Unsupervised Anomaly Detection Methods

**Figure 7:**
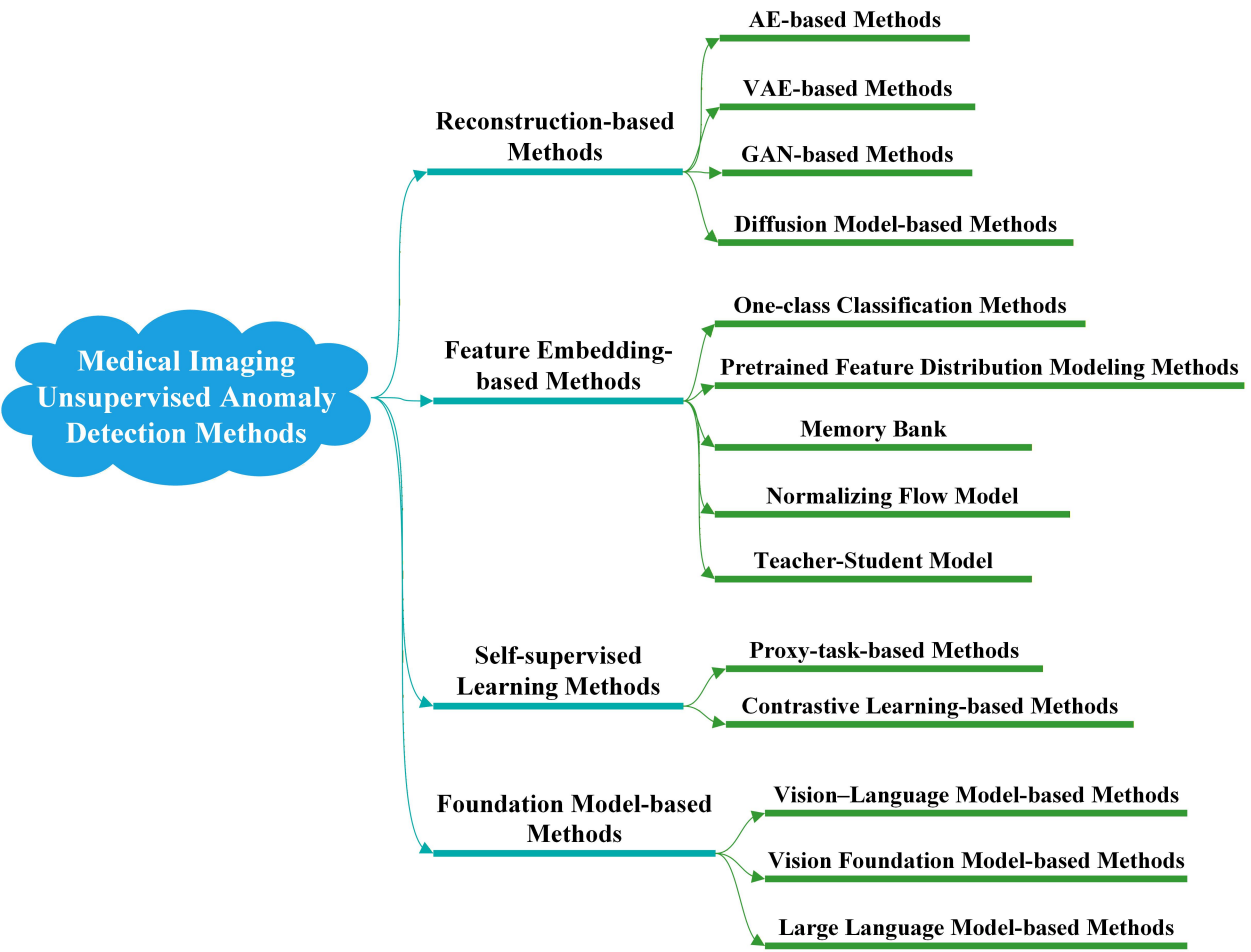
Taxonomy of medical imaging unsupervised anomaly detection (MUAD) methods. Hybrid methods are placed according to their dominant anomaly modeling mechanism, while auxiliary components are discussed in the corresponding sections.

This section provides a systematic review of representative methods for MUAD. As illustrated in Figure 7, existing MUAD approaches can be broadly categorized into four groups: reconstruction-based methods, feature embedding-based methods, self-supervised learning methods, and foundation model-based methods.

Given that many recent MUAD methods integrate multiple technical elements, such as hybrid frameworks that combine reconstruction-based modeling with self-supervised learning, or foundation model-based approaches that introduce proxy tasks to construct auxiliary anomaly cues, this survey adopts a dominant paradigm-oriented principle for method categorization. Specifically, each method is assigned to the category corresponding to the core modeling idea that most directly determines how normal patterns are learned and how anomaly scores are generated. This principle is adopted because it reflects the core logic of MUAD methods: the way normality is modeled determines what the method regards as a reference for normal patterns, while the way anomaly evidence is derived determines how deviations are ultimately quantified and interpreted. Concretely, the categorization in this survey is mainly based on the following three aspects:

1. Which modeling paradigm the core methodological innovation of the paper primarily contributes to.
2. Which mechanism is mainly used to model the distribution of normal data.
3. Which module serves as the primary source of anomaly evidence or anomaly scores.

For hybrid methods, we assign them to the category that plays the dominant role in anomaly detection, while secondary components are briefly discussed where appropriate. This helps reduce ambiguity in categorizing hybrid methods and allows their main modeling contribution to be understood more clearly. Overall, this strategy helps avoid excessive fragmentation caused by differences in implementation-level module combinations, thereby providing a clearer view of the fundamental modeling logic, major strengths and limitations, and overall development trajectory of different MUAD methods. More importantly, it helps readers better understand the core modeling logic of different MUAD methods, compare their strengths and limitations under a unified perspective, and follow the overall development of the field more clearly.

### 3.1. Reconstruction-Based Methods

Reconstruction-based unsupervised anomaly detection methods represent one of the earliest and most fundamental paradigms in MUAD. The core idea is to learn the encoding process, latent space representation, and decoding mechanism of normal images only. During inference, abnormal regions cannot be faithfully reconstructed, leading to significant reconstruction discrepancies that can be used for anomaly detection.

**Figure 8:**
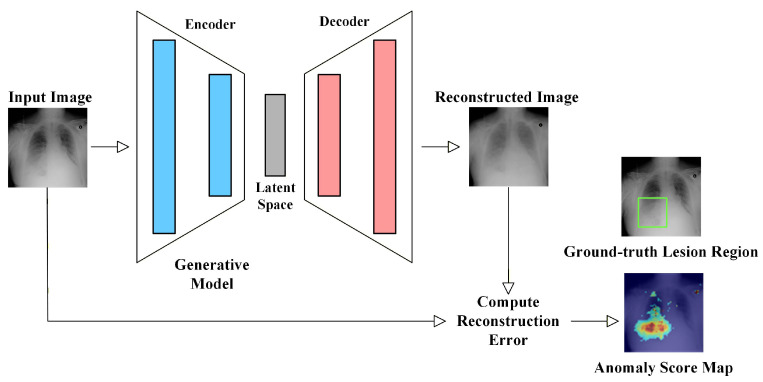
Illustration of reconstruction-based methods. The generative model can be implemented using AE, VAE, GAN, or diffusion models, and anomalies are detected based on reconstruction errors. High-intensity regions in the anomaly map indicate potential abnormal lesions, while the green bounding box denotes the ground-truth lesion region.

As illustrated in Figure 8, the input image is reconstructed into a pseudo-normal version, and the difference between the input and reconstructed image can be used to localize potential lesions. Depending on the type of generative model employed, existing reconstruction-based methods can be further divided into AE-based, VAE-based, GAN-based, and diffusion-based approaches.

#### 3.1.1. Autoencoder-Based Methods

AEs are among the earliest reconstruction-based methods applied to MUAD. They encode input images into latent representations through an encoder and reconstruct the images via a decoder. Due to the relatively stable structural priors in medical images (e.g., brain MRI, chest X-ray, and retinal images), AEs can effectively capture the consistency of normal anatomical structures. Early studies, such as those by Zimmerer et al. [102] and Baur et al. [103], demonstrated that convolutional AEs can reconstruct normal anatomical structures in brain MRI and highlight abnormal regions via reconstruction residuals. However, such models typically suffer from two major limitations: (1) Identity mapping problem: due to strong model capacity, the network may learn to directly copy the input image instead of reconstructing a normalized version, resulting in the so-called anomaly leakage, where abnormal regions are also reconstructed. (2) Blurry reconstruction: deep AEs often fail to preserve high-frequency texture details, leading to blurred reconstruction even in normal regions.

To improve robustness, denoising autoencoders (DAE) [104] have been introduced. By adding noise to normal images and learning to recover clean images, DAE encourages the model to focus on structural information rather than pixel-wise replication. Kascenas et al. [85,105] systematically analyzed the impact of noise patterns on DAE performance and showed that coarse-grained noise at intermediate spatial scales is more effective than pixel-level noise in enforcing structural consistency, thereby improving anomaly sensitivity. With the increasing adoption of Transformer architectures, masking strategies have also been introduced to mitigate the identity mapping problem. Tian et al. [106] employed a high ratio of random masking, forcing the model to reconstruct missing regions based on global contextual information rather than relying solely on visible patches. Additionally, multi-level cross-attention mechanisms were introduced in the decoder to fuse hierarchical semantic features, improving reconstruction consistency. In three-dimensional scenarios, AE-based methods have also been extended to volumetric modeling. Luo et al. [107] proposed a 3D residual AE framework that directly reconstructs whole-brain MRI volumes, enabling the model to capture spatial relationships across slices.

Another line of research focuses on improving the structural consistency of AE-based models. Zhang et al. [108] pointed out that skip connections may introduce semantic inconsistencies between encoder and decoder features, thereby weakening anomaly discrimination. To address this issue, they designed chained convolutional blocks for feature alignment and incorporated adversarial latent constraints to enhance reconstruction quality. Bercea et al. [109] explicitly modeled local deformation fields to align anatomical variations across individuals, while incorporating perceptual loss to preserve global structural consistency. This approach effectively mitigates the tendency of shallow AEs to reconstruct anomalies and deep AEs to produce overly smooth reconstructions. Lu et al. [35] proposed a heterogeneous architecture with a CNN-based encoder and a hybrid CNN–Transformer decoder, leveraging different inductive biases to avoid identity mapping. Furthermore, they replaced pixel-wise reconstruction errors with multi-stage feature comparison, thereby amplifying reconstruction discrepancies in anomalous regions. Bercea et al. [110] further introduced a reverse training mechanism, incorporating multi-scale consistency loss and adversarial latent constraints to improve reconstruction quality. Their method demonstrates robust performance across multiple modalities, including MRI, chest X-ray, and wrist X-ray.

Notably, Cai et al. [111] showed that the effectiveness of AE-based anomaly detection is closely related to the capacity of the latent space. An excessively large latent space tends to encourage identity mapping, allowing anomalies to be reconstructed. In contrast, a properly constrained latent space forces the model to preserve normal patterns while suppressing abnormal features. This finding provides an important guideline for the structural design of AE-based models.

#### 3.1.2. Variational Autoencoder-Based Methods

VAEs extend standard autoencoders by introducing probabilistic modeling, where input images are mapped to latent distributions rather than deterministic feature vectors. By imposing a Kullback–Leibler (KL) divergence constraint on the latent space, VAEs learn a continuous, structured, and semantically meaningful distribution of normal samples. In the context of unsupervised anomaly detection, VAEs can identify anomalies using both reconstruction errors and deviations in latent distributions. However, this mechanism also introduces certain limitations. The KL regularization term encourages the latent space to approximate an isotropic Gaussian distribution, while the reconstruction process typically relies on an $\ell_{2}$ loss. Together, these factors often lead to over-smoothed reconstructions with blurred edges and loss of fine details. Early work by Zimmerer et al. [112] systematically compared reconstruction error, KL divergence, and ELBO gradients for anomaly localization, demonstrating that combining KL gradients with reconstruction errors significantly improves the stability of brain tumor localization. Importantly, they highlighted that latent distribution shift itself serves as a critical cue for anomaly detection.

To enhance structural representation, Marimont and Tarroni [77] proposed using implicit field representations instead of direct pixel-wise modeling, resulting in sharper reconstructions and alleviating the blurring issue inherent in conventional VAEs. In addition, vector quantized VAE (VQ-VAE) [113] introduces discrete latent representations, strengthening latent space constraints. Marimont et al. [72] further discretized the latent space into a visual codebook, forcing images to be projected onto normal patterns. They also proposed a latent repair strategy, which projects anomalous inputs back into the normal latent space during inference, yielding more reliable reconstructions and improving anomaly localization performance in brain MRI. Pinaya et al. [24] combined VAE with 3D Transformer architectures to enhance cross-slice semantic modeling through attention mechanisms. By incorporating an autoregressive prior, their approach improves the structural organization of the latent space and achieves better global consistency in 3D brain MRI reconstruction.

To improve clinical robustness, StRegA [81] replaces raw MRI inputs with brain tissue segmentation maps, reducing cross-center variability. Building upon ceVAE (context-encoding VAE) [102], it further constructs a more compact encoding structure, enabling the model to learn normal anatomical distributions within a more consistent structural space. Tri-VAE [114] points out that conventional projection-based VAEs cannot guarantee alignment between anomalous inputs and the normal latent space. To address this, it introduces triplet metric learning to compress the distribution of normal samples in latent space and employs a resampling strategy for anomaly alignment. Additionally, gated skip connections are used to suppress anomaly leakage, further improving anomaly detection performance.

#### 3.1.3. GAN-Based Methods

GANs learn the distribution of normal samples through adversarial training between a generator and a discriminator. The generator produces realistic normal images from latent variables, while the discriminator distinguishes between real samples and generated outputs. Compared with AEs and VAEs, GAN-based methods are more effective at capturing high-frequency textures and fine structural details, making them particularly suitable for medical imaging tasks with strong structural priors. However, GAN training relies on adversarial optimization, which is inherently unstable and prone to mode collapse, where the generator learns only a subset of the normal distribution. This often results in limited diversity in generated images and may cause abnormal structures to be incorrectly reconstructed as normal patterns.

AnoGAN [54] is one of the earliest methods that applied GANs to reconstruction-based anomaly detection in medical imaging. It performs anomaly detection by iteratively optimizing latent variables so that the generated image matches the input as closely as possible. However, this process is computationally expensive and results in slow inference. f-AnoGAN [71] improves efficiency by introducing an encoder to directly estimate latent representations, but it still struggles to capture fine-grained details, leading to suboptimal reconstructions. Considering that structural abnormalities often exhibit continuity across slices, Han et al. [73] introduced multi-slice reconstruction by leveraging contextual information from adjacent MRI slices, thereby improving sensitivity to structural anomalies. Kuttala et al. [115] further incorporated dense connections and attention mechanisms to enhance the modeling of local structural variations. In clinical applications, Kim et al. [116] demonstrated that combining reconstruction error with feature distance enables effective anomaly scoring for CT images in breast radiotherapy patients, highlighting the practical applicability of GAN-based reconstruction methods.

As research progresses, GAN-based methods have evolved from simply reconstructing normal images to generating lesion-free images while preserving individual anatomical structures [117]. The key idea is to maintain patient-specific anatomy while repairing lesion regions to resemble normal tissue, thereby producing clearer anomaly boundaries in difference maps. For example, Bercea et al. [118] proposed a two-stage inverse generation strategy: first estimating suspicious regions in latent space and then performing localized normal restoration using GAN. This forward–backward mapping inconsistency enhances anomaly localization accuracy. More recent approaches attempt to leverage abundant unlabeled real-world data [25,80,88,119], thereby improving generalization and reducing reliance on purely normal datasets. Instead of training solely on normal samples, these methods jointly use normal and unlabeled images as input and enforce reconstruction into the normal domain, effectively enlarging the discrepancy between normal and abnormal samples in reconstruction space. For instance, HealthyGAN [80] employs mask-guided implicit cycle consistency to generate pseudo-normal images. Brainomaly [25] focuses on aligned T1 MRI data and enhances anomaly detection by learning local residual maps that preserve structure while highlighting abnormalities. Zhang et al. [88] introduced anatomical-consistent positional encoding and hierarchical attention gating mechanisms to improve lesion restoration. PAttL-GAN [119] further incorporates multi-scale fusion and pixel-level attention, making reconstructed pseudo-normal images more sensitive to local structural differences.

In addition, Bougaham et al. [120] utilized cycle-consistent adversarial networks to enforce bidirectional constraints between normal and abnormal domains, while incorporating structure-preserving loss to maintain anatomical consistency during lesion correction. To alleviate the identity mapping problem, Zhang et al. [32] proposed dynamically disabling skip connections to prevent direct copying of low-level textures, thereby enhancing the distinguishability of anomalous regions. Furthermore, they introduced histogram of oriented gradients (HOG) as an auxiliary structural feature and adopted multi-task learning to improve sensitivity to local edge and texture variations.

#### 3.1.4. Diffusion Model-Based Methods

Diffusion models learn the distribution of normal samples by progressively adding noise to images and restoring them through a reverse denoising process. During inference, the model maps an input image to its pseudo-normal counterpart, and anomalies are revealed through reconstruction discrepancies. Compared with GAN-based approaches, diffusion models offer more stable training and are not affected by mode collapse. AnoDDPM [55] is the first work to introduce diffusion models into MUAD. It replaces conventional Gaussian noise with simplex noise to more effectively disrupt low-frequency structures and adopts a partial diffusion strategy to significantly reduce inference cost, enabling its application to high-resolution medical images. While AnoDDPM improves pseudo-normal generation from the perspective of noise design, Wolleb et al. [10] focused on the diffusion process itself and proposed multi-step reconstruction residuals for anomaly localization, systematically analyzing key design factors of diffusion-based methods.

However, performing global diffusion over entire images is computationally expensive and often lacks sensitivity to small or subtle anomalies. As a result, subsequent research has shifted toward more efficient local modeling strategies. For example, pDDPM [121] applies noise only to image patches and reconstructs them using contextual information from neighboring regions, thereby enhancing local detail modeling while preserving global consistency. mDDPM [82] further introduces higher masking ratios to encourage the model to focus on structural reconstruction rather than texture recovery, leading to more stable anomaly localization. Bercea et al. [23] proposed an automatic mask generation strategy combined with local lesion restoration, and employed multiple resampling mechanisms to improve robustness and generalization. Beizaee et al. [122] extended this idea by performing masked diffusion in latent space and applying selective reverse diffusion to predicted anomalous regions during inference, enabling targeted local restoration and more precise lesion localization.

Meanwhile, another line of work emphasizes modeling global structural consistency. Behrendt et al. [123] extended diffusion-based reconstruction to 3D volumetric data and replaced pixel-wise $\ell_{1}/\ell_{2}$ residuals with structure consistency scores, improving the reliability of 3D lesion separation. Bercea et al. [124] introduced an implicit guidance mechanism that dynamically integrates temporal anomaly maps across diffusion steps, encouraging more complete restoration in abnormal regions while preserving appearance consistency in normal areas. Building upon this, Fontanella et al. [89] incorporated counterfactual generation into diffusion models by using prior saliency maps as intervention masks. Their approach applies stochastic denoising in anomalous regions and deterministic denoising in normal regions, forming a dual-path sampling strategy that repairs lesions while preserving individual anatomical structures. Compared with pseudo-normal reconstruction, the resulting counterfactual images exhibit stronger anatomical consistency and clearer lesion boundaries, improving interpretability. Bi et al. [125] proposed a structured noise injection strategy and multi-stage diffusion reconstruction, enabling coarse-to-fine pseudo-normal generation and producing more stable and distinct anomaly maps. In more recent work, Kim et al. [28] combined diffusion models with medical knowledge by incorporating electronic health record (EHR) information as cross-modal semantic guidance. Additionally, a checkerboard masking strategy was employed to enhance the consistency of pseudo-normal reconstruction in chest X-ray images, improving adaptability to individual variations and structural heterogeneity.

#### 3.1.5. Summary of Reconstruction-Based Methods

Reconstruction-based methods constitute the earliest and one of the most fundamental paradigms in MUAD. Among them, AE and VAE models are relatively simple but have limited capacity to capture fine-grained lesions. GAN-based methods are capable of modeling high-frequency textures but suffer from training instability. Diffusion models achieve the highest reconstruction fidelity but are computationally expensive and, without proper constraints, may over-reconstruct anomalous regions.

One of the central challenges of reconstruction-based methods is the identity mapping problem. Since the training objective primarily minimizes reconstruction error, models tend to directly replicate the input image, treating anomalies as normal variations and reconstructing them accordingly. This issue becomes more pronounced as model capacity increases. However, excessively restricting reconstruction capability may prevent the generation of reliable pseudo-normal images. Therefore, reconstruction-based methods face an inherent trade-off between accurately restoring normal structures and effectively amplifying anomaly discrepancies. Although reconstruction residuals or structural consistency scores can serve as anomaly indicators, clinical diagnosis requires more explicit anatomical and semantic interpretations. Existing reconstruction-based approaches still lack sufficient clinical interpretability.

From a developmental perspective, reconstruction-based MUAD methods exhibit three notable trends: (1) A shift from global image reconstruction to localized and semantically informed reconstruction. Approaches such as masked reconstruction, patch-wise diffusion, and latent space repair no longer aim to recover the entire image, but instead focus on generating anatomically plausible normal structures. (2) A transition from generic image generation to the construction of clinically meaningful normal reference images. Many recent studies explicitly incorporate medical knowledge—such as anatomical structures, imaging physics, spatial priors, and electronic health records—into pseudo-normal generation, making the results more consistent with clinical reasoning. (3) An evolution from single reconstruction objectives to multi-task integration, where reconstruction is jointly modeled with feature embedding, contrastive learning, attention mechanisms, and memory modules to achieve more robust normal pattern modeling. Recent studies [3] further indicate that relying solely on pixel-level reconstruction is insufficient for complex medical imaging tasks. Future reconstruction-based MUAD methods should therefore focus on capturing anatomical semantics and generating outputs that better align with real clinical scenarios.

### 3.2. Feature Embedding-Based Methods

Different from reconstruction-based approaches that rely on pixel-level reconstruction errors, feature embedding-based anomaly detection focuses on modeling the statistical structure of normal patterns in deep semantic feature spaces. These methods construct a stable normal feature space and quantify anomalies based on the degree of feature deviation. According to different feature modeling strategies, existing approaches can be broadly categorized into one-class classification methods, pre-trained feature distribution modeling methods, memory-based methods, normalizing flow models, and teacher–student frameworks.

#### 3.2.1. One-Class Classification Methods

One-class classification (OCC) methods aim to learn a decision boundary that encloses the majority of normal samples in the feature space, and measure anomaly scores based on the deviation of a sample from this boundary or region. Classical approaches include One-Class Support Vector Machine (OC-SVM) [126] and Support Vector Data Description (SVDD) [127]. The former learns a hyperplane in a high-dimensional space using kernel functions to separate data from the origin, while the latter models normal data as a compact hypersphere with minimal enclosing volume. With the development of deep learning, DeepSVDD [128] and its variants extend this idea to deep embedding spaces, enabling end-to-end training that encourages normal samples to cluster within a hyperspherical region in latent space. Liznerski et al. [129] further introduced anomaly score maps to enhance interpretability, making the approach more applicable in practical scenarios. To address fine-grained anomaly detection, Patch SVDD [75] extends SVDD to patch-level feature modeling, improving the capability of capturing local anomalies. Nguyen et al. [130] applied Gaussian interpolation to smooth feature distributions, thereby enhancing cross-modality stability. In specific medical applications, such as COVID-19 CT screening, Alhadad et al. [131] combined DeepSVDD with transfer learning to improve model adaptability. Meta-SVDD [132] further introduces meta-learning to enable few-shot adaptation across different pathological tasks. Despite their clear theoretical formulation and structural simplicity, OCC methods face limitations in modeling the highly complex and heterogeneous distributions of medical images. Due to significant inter-patient variability and intricate anatomical structures, normal patterns are difficult to represent as compact regions in feature space, which restricts the applicability of OCC methods in modern MUAD scenarios.

#### 3.2.2. Pre-Trained Feature Distribution Modeling Methods

Pre-trained feature distribution modeling methods utilize deep features extracted from networks such as ResNet [133] and WideResNet [134], which are pre-trained on ImageNet [135], to learn the statistical regularities of normal samples in the feature space of medical images. Compared with early one-class boundary-based methods, pre-trained features inherently capture richer semantic representations, making them more suitable for modeling structural consistency across different scales and anatomical regions.

Ouardini et al. [136] were among the first to apply pre-trained feature similarity for anomaly detection in retinal images. PaDiM [58] extracts multi-scale features and models them using multivariate Gaussian distributions, where Mahalanobis distance is used to measure feature deviations, and has become a representative approach in this category. Wang et al. [137] proposed a local–global semantic consistency strategy to identify deviations at different semantic levels. SPADE [138] performs pixel-wise nearest neighbor matching based on multi-scale feature pyramids, enhancing the localization of fine-grained structural anomalies. Das et al. [33] combined domain-specific pre-trained features with sparse dictionary learning to construct a multi-scale sparse coding framework, achieving robust anomaly detection in retinal and OCT images. ADFA [41] employs WideResNet to extract multi-level features and improves the compactness of normal feature distributions through attention mechanisms and differentiable top-*k* selection. González-Jiménez et al. [139] modeled pre-trained features in hyperbolic space and constructed weighted semantic centroids to enhance separability among anomaly categories. Xu et al. [90] identified issues of ambiguous mapping and misaligned mapping when transferring pre-trained features to medical images, and proposed a semantic alignment mechanism based on intra- and inter-sample correlations to improve robustness. Tang et al. [83] pointed out that directly freezing ImageNet encoders leads to a semantic gap, and introduced a stop-gradient strategy to improve feature adaptation in the medical domain. The main contribution of pre-trained feature distribution modeling methods is the establishment of a paradigm that models normal patterns in a structured feature space. This paradigm provides the basis for subsequent developments, including memory-based modeling, normalizing flow methods, and teacher–student frameworks.

#### 3.2.3. Memory-Based Methods

To more stably characterize the diversity of normal tissues, researchers construct memory banks in the pre-trained feature space to compactly store feature templates or prototypes of normal distributions. A prototype refers to a feature vector that represents a typical normal structure or texture pattern, serving as an important reference during inference. Since memory banks provide stable normal templates, they are often combined with reconstruction models to suppress the direct reconstruction of anomalies.

**Figure 9:**
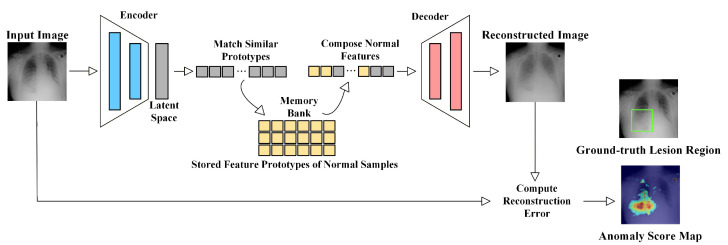
Illustration of memory bank and reconstruction model-based methods. Input features retrieve the most similar normal prototypes from the memory bank for reconstruction. Regions that cannot be matched with normal prototypes are highlighted as anomalies in the anomaly score map.

As illustrated in Figure 9, such methods first map the input into a latent representation, retrieve the most similar normal prototypes from the memory bank, and reconstruct the image based only on these prototypes. Consequently, anomalous regions are difficult to reconstruct accurately. When input features fail to find sufficiently similar prototypes in the memory bank, they are considered anomalous. MemAE [60] first introduced normal feature memory units into autoencoders, restricting reconstruction to rely on a limited number of normal templates and thus reducing anomaly leakage. Park et al. [140] further proposed a read-only memory mechanism, which retrieves the most similar patches from the normal memory via attention. Different from methods relying on image reconstruction and pixel-level errors, PatchCore [59] constructs a compact prototype set by selecting representative subsets from the feature distribution and performs efficient patch-level anomaly localization based on nearest neighbor distances.

With the development of pre-trained vision models and multi-scale feature modeling, memory-based methods have gradually adapted to the multi-scale and cross-anatomical characteristics of medical images. Chen et al. [76] combined attention and memory modules on multi-scale features to capture both local textures and global anatomical patterns. Ceauşescu et al. [141] proposed an adaptive feature compression strategy to better cover inter-patient variability. Tian et al. [106] embedded multi-level memory modules and cross-layer attention into masked autoencoders, encouraging the reconstruction of normal structures while suppressing anomalous features. Kim et al. [29] constructed a 3D structural memory bank from multi-view projections in chest CT, improving cross-slice consistency in low-dose imaging. More recent studies focus on structure consistency and semantic alignment for memory modeling. SimSID [19] learns structured memory by aligning anatomical regions such as lungs, mediastinum, and cardiac silhouettes, leading to clearer normal patterns in chest X-ray features. Xu et al. [90] introduced intra- and inter-sample correlation constraints to perform semantic purification before features are stored in the memory bank. Ye et al. [142] proposed a dual-memory structure to separately model local textures and global structures, enabling more fine-grained representations.

The development of memory-based methods has also promoted prototype-driven interpretability. Cho et al. [143] constructed class-level prototype sets and identified anomalies when local features fail to match any normal prototypes, generating explanation maps based on prototype inconsistency. Wei et al. [22] extended memory banks into the latent space of GANs and performed coarse-to-fine retrieval with local latent replacement for pseudo-normal reconstruction, significantly improving anomaly localization in brain MRI. Huang et al. [46] proposed an uncertainty-aware prototype learning framework using Transformer-based memory and pixel-wise Bayesian uncertainty modeling. SP-Mamba [144] further emphasizes spatially-aware prototypes, learning reliable local prototypes via sliding windows and combining them with efficient multi-scale modeling. Overall, memory-based methods explicitly construct a reference space of normal features, enabling retrieval and comparison of normal patterns, and thus better accommodating the diversity of medical images across patients and anatomical structures. However, these methods typically rely on large-scale feature repositories and nearest neighbor search, which may introduce challenges in computational efficiency and memory consumption.

#### 3.2.4. Normalizing Flow-Based Methods

**Figure 10:**
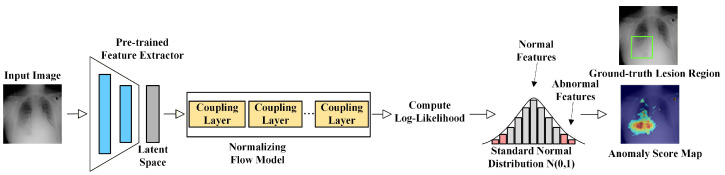
Illustration of normalizing flow based methods. Normalizing flows construct invertible mappings using multiple coupling layers, where conditional transformations are applied to part of the features to enable efficient bidirectional density transformation and log-likelihood computation. Regions with low likelihood are identified as anomalies in the anomaly score map.

Normalizing flow (NF) models map complex data distributions to simple prior distributions (typically Gaussian) through a series of invertible transformations, which are usually implemented by stacking multiple coupling layers. During inference, normal samples tend to have higher likelihoods in the latent space, while anomalous samples exhibit lower likelihood due to deviations from the learned distribution. Therefore, likelihood deviation naturally serves as an important anomaly score. As illustrated in Figure 10, NF models perform invertible mappings of latent features and use log-likelihood as the anomaly metric. DifferNet [74] was among the first to introduce NF into image anomaly detection. However, due to limitations in gradient propagation and shallow feature representations, it shows limited capability in capturing structural anomalies. CFLOW [79] further improves stability by extracting multi-scale features using pre-trained convolutional pyramids and estimating their likelihoods via flow models. CS-FLOW [61] extends single-scale flow modeling into a cross-scale invertible structure, enabling collaborative modeling of hierarchical features. FastFlow [62] preserves spatial correspondence through a two-dimensional flow structure, although its relatively shallow architecture limits representation capacity.

To better adapt NF models to medical imaging scenarios, recent studies incorporate modality-specific designs. Valiuddin et al. [145] embedded wavelet transforms into flow models to perform multi-scale density modeling in the frequency domain, enhancing sensitivity to fine-grained texture anomalies in dermoscopic images. Zhao et al. [38] combined NF with reconstruction frameworks by embedding lightweight flows into autoencoders, and proposed a joint anomaly score based on reconstruction error and flow likelihood, improving detection of low-contrast lesions. Although NF models still face challenges in training stability and computational efficiency in high-dimensional feature spaces, their ability to provide explicit probabilistic modeling and interpretable likelihood-based anomaly scores makes them a valuable approach for improving the reliability and interpretability of MUAD.

#### 3.2.5. Teacher–Student Models

**Figure 11:**
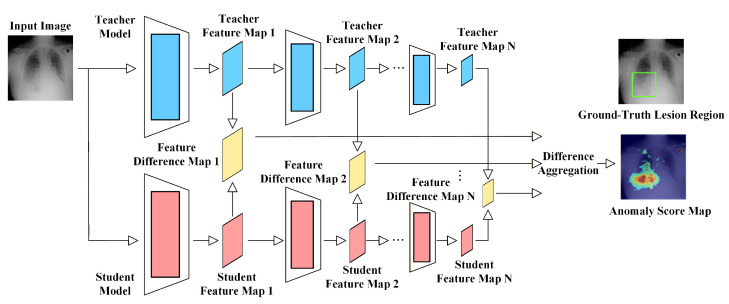
Illustration of the teacher–student framework. The student network is trained to mimic the normal feature representations learned by the teacher network. Feature discrepancies between the teacher and student at multiple layers are computed to produce feature difference maps, which are subsequently aggregated to generate the anomaly score map.

The core idea of the teacher–student (TS) framework is to train a student network to mimic the multi-scale feature representations of a pre-trained teacher network using only normal samples. When anomalous medical images are input, the student network fails to accurately reproduce the normal feature patterns of the teacher across different layers, and the resulting feature discrepancy is used as the anomaly score. The overall framework is illustrated in Figure 11. This paradigm was first proposed by Bergmann et al. [146], who demonstrated that, in the absence of anomalous samples, the student network can only learn structural characteristics of normal data, and thus naturally deviates in anomalous regions. Subsequently, Salehi et al. [57] introduced a multi-resolution distillation mechanism to enhance both local and global semantic consistency, and validated its effectiveness in medical imaging. Rudolph et al. [78] further performed cross-stage distillation using feature pyramid matching, while Deng et al. [56] proposed reverse distillation, where the student network has higher capacity and stronger representation ability than the teacher. Since a stronger student is required to approximate a weaker teacher’s normal feature mapping, anomalous regions—being inherently harder to mimic—tend to produce amplified deviations.

In medical scenarios, teacher–student models are particularly effective due to their sensitivity to multi-scale structural consistency. Liu et al. [147] introduced skip connections and multi-scale consistency constraints to enforce structural alignment across different receptive fields, thereby improving sensitivity to small lesions and irregular boundaries in lung images. Wang et al. [148] focused on the layered structure of OCT and applied layer-wise distillation to enhance the model’s sensitivity to retinal boundaries, enabling more precise localization of subtle thickness variations and local disruptions. Reverse distillation is especially suitable for medical tasks, as most lesions disrupt local anatomical structures, which are difficult for the student network to replicate. Based on this observation, Ge et al. [36] proposed an improved direct reverse distillation strategy by enhancing skip connections to suppress pseudo-consistency in anomalous regions, leading to sharper anomaly responses at structural boundaries. Li et al. [98] further introduced scale-aware contrastive distillation, leveraging cross-scale positive and negative pairs to enhance feature deviation at anomalous regions while simultaneously handling both large tumors and small infarctions. Dong et al. [94] combined teacher–student distillation with student self-distillation between support and query samples, and introduced a dynamic weighting strategy to adaptively evaluate the relevance of support samples, thereby improving generalization in few-shot medical anomaly detection.

For 3D medical anomaly detection tasks, teacher–student models have been further extended. Schwarz et al. [149] proposed a 3D patch-level pyramid distillation framework to maintain structural continuity across slices in MRI, addressing the limitations of 2D distillation in capturing volumetric consistency. Liu et al. [30] introduced aggregated masked convolution and centralized linear attention to dynamically filter potential anomalous regions during distillation, enhancing multi-scale structural modeling in CT images. Rahmaniar et al. [150] incorporated domain-invariant and domain-specific feature decomposition and aligned teacher and student feature distributions using adversarial learning, achieving stable performance across multiple datasets. Li et al. [49] combined graph neural networks with teacher–student models, enabling node-level feature alignment to preserve structural consistency across regions in large-scale histopathological images. Overall, by leveraging cross-layer feature imitation and deviation aggregation, teacher–student models significantly enhance anomaly saliency while maintaining model efficiency. They have become an important approach in MUAD, although their anomaly discrimination capability is still influenced by the representation capacity of the teacher network.

#### 3.2.6. Summary of Feature Embedding-Based Methods

Feature embedding-based methods in MUAD measure anomalies by constructing a normal feature space and quantifying deviations within it. Compared with reconstruction-based approaches, they offer advantages in stability and semantic representation. However, several key challenges remain in real-world medical scenarios. First, most methods rely on encoders pre-trained on natural images, which introduces a semantic gap between learned representations and medical tasks. This often leads to feature misalignment or mismatch, making it difficult to accurately model normal feature distributions. Second, many approaches adopt Gaussian modeling, memory banks, or flow-based methods to describe feature distributions. However, due to the intrinsic variability of normal tissues in medical images, feature distributions are often non-spherical, non-Gaussian, and even multi-modal, making it difficult for a single distribution assumption to fully capture their complexity.

From a developmental perspective, feature embedding-based methods are evolving from static normal feature modeling toward dynamic semantic alignment and structural consistency modeling. On one hand, increasing efforts focus on feature purification, domain alignment, and structural guidance before entering the feature space, making normal feature distributions more compact and stable at the semantic level. On the other hand, research is gradually shifting from single-scale feature deviation detection to multi-level and cross-modal semantic fusion. By incorporating mechanisms such as cross-scale feature aggregation, cross-sequence consistency learning, knowledge distillation, and medical prompt learning, models are able to more precisely capture both fine-grained local anomalies and global structural deviations. In the future, feature embedding-based methods are expected to further emphasize medical domain-specific representation, multi-modal knowledge integration, and structured distribution modeling, in order to achieve improved robustness, interpretability, and clinical applicability.

### 3.3. Self-Supervised Learning Methods

Self-supervised learning (SSL) guides models to learn stable structural and semantic representations under unlabeled conditions by constructing auxiliary tasks or feature consistency constraints, thereby indirectly enhancing their ability to perceive semantic deviations of anomalies. Given the scarcity of anomaly annotations and the diversity of anomaly patterns in medical imaging, SSL has gradually become an important representation learning paradigm in MUAD. This section introduces its modeling strategies and adaptations to medical scenarios from two perspectives: proxy task-based methods and contrastive learning-based methods.

#### 3.3.1. Proxy Task-Based Methods

**Figure 12:**
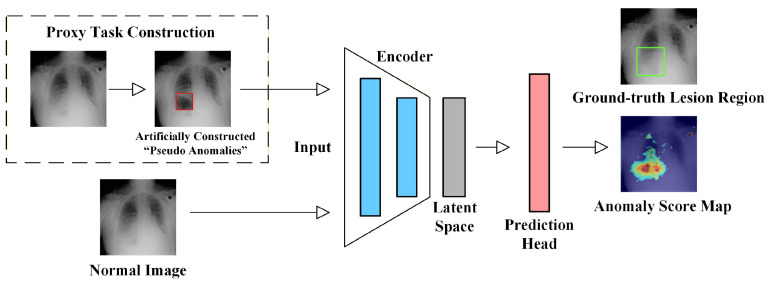
Illustration of proxy task-based methods for medical imaging unsupervised anomaly detection. Pseudo anomalies are artificially injected during training to enable the model to learn normal structural patterns. During inference, the model can therefore identify real anomalies. The example illustrates a local pseudo-anomaly injection proxy task.

Proxy task-based SSL methods transform anomaly detection into trainable surrogate tasks by constructing artificial pseudo-supervision signals. As illustrated in Figure 12, the core idea is to apply structural perturbations to normal images, enabling the model to learn implicit normal semantic patterns through recovering or recognizing these perturbations, and thus automatically detect real anomalies during inference. Early methods such as FPI [65], PII [66], and NSA [64] adopt tasks such as image restoration, occlusion completion, and noise prediction to learn contextual relationships of normal patterns. CutPaste [63] generates pseudo anomalies by cutting and pasting random regions within normal images, allowing the model to learn sensitivity to local anomalies via a binary classification proxy task. DRÆM [151] further adopts a dual-branch structure combining pseudo anomaly generation and reconstruction consistency, enhancing the model’s response to subtle defects, and has influenced subsequent extensions in medical imaging.

As SSL methods are adapted to medical scenarios, researchers have recognized that proxy tasks should preserve anatomical plausibility; otherwise, pseudo anomalies may disrupt real medical semantics and degrade performance. AnatPaste [152] constrains pseudo anomalies within specific anatomical regions in chest X-rays, enabling the model to learn more realistic structural deviations. AMAE [20], based on masked autoencoders (MAE), employs large-scale masking reconstruction to learn multi-scale normal priors and enhances sensitivity to structural anomalies through dual-distribution modeling. Li et al. [87] further integrate proxy tasks into reconstruction frameworks by introducing BYOL-style self-supervised modules [153], enabling the learning of multi-granularity semantic priors such as color, texture, and layered structures. Different augmentation strategies correspond to different types of anomalies (e.g., color shifts or structural disruptions), thereby improving the saliency of reconstruction residuals. DDAD-ASR [84] applies SSL to refine anomaly scores rather than directly detect pseudo anomalies, generating initial anomaly responses through dual-distribution modeling and improving localization accuracy and robustness via a self-supervised refinement network.

To better simulate structural variations in medical images, recent studies design proxy tasks based on morphology and boundary characteristics. Deng et al. [154] propose random-shaped pseudo outliers to mimic lesion-like boundary discontinuities and structural irregularities, improving the model’s ability to learn anomaly morphology. In more complex anatomical scenarios, proxy tasks are increasingly designed in a multi-task manner. Baugh et al. [155] combine multiple tasks such as rotation prediction, jigsaw puzzle solving, and strong–weak augmentation consistency, enabling the model to capture texture, spatial arrangement, and global consistency simultaneously. While these methods effectively learn structural semantics and contextual relationships without annotations, their performance depends on the design of pseudo anomaly generation, and the semantic gap between pseudo anomalies and real lesions may still limit detection performance.

#### 3.3.2. Contrastive Learning-Based Methods

**Figure 13:**
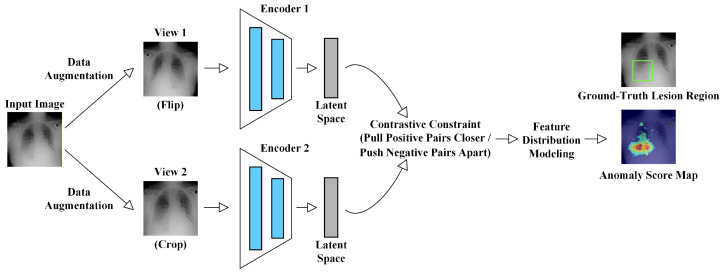
Illustration of the contrastive learning framework. Multiple augmented views (e.g., flipping and cropping) are generated from the input image to construct instance-level contrastive constraints. The model learns discriminative feature representations by pulling positive pairs closer and pushing negative pairs apart in the latent space. Some implementations employ a shared encoder to learn consistent feature representations for anomaly detection.

Contrastive learning learns discriminative representations through instance-level similarity constraints, with representative frameworks including SimCLR [156], MoCo [157], and SimSiam [158], which does not require explicit negative samples. The core idea is to enforce consistency between different views of the same instance, such that anomalous regions—being difficult to maintain semantic consistency—naturally deviate in feature space. As illustrated in Figure 13, contrastive learning compresses normal distributions through multi-view consistency constraints, while anomalies exhibit significant deviations due to semantic inconsistency. In MUAD, contrastive learning methods are adapted by tailoring positive and negative sample construction as well as semantic constraint granularity to better capture structural and semantic differences in medical images. Early studies primarily utilize contrastive learning as a feature enhancement strategy. Spahr et al. [159] employ SimCLR-style pretraining to learn global morphological representations of normal samples via cross-sample similarity constraints, resulting in more compact embeddings and improved anomaly discrimination. PMSACL [47] introduces a pseudo multi-class contrastive pretraining strategy, where different augmentations of the same image are treated as distinct pseudo classes, forcing the model to learn boundaries between structure-preserving and structure-disrupting transformations.

Considering that medical anomalies often manifest as local semantic shifts rather than simple appearance changes, some studies impose contrastive constraints at a finer granularity. CCD [68] extends contrastive learning from instance-level comparison to distribution-level comparison, enabling the model to explicitly separate normal and anomalous feature distributions under unlabeled conditions, which is particularly suitable for detecting fine-grained structural anomalies. CRADL [86] proposes medically consistent augmentation strategies (e.g., preserving anatomical structure, avoiding unrealistic color perturbations, and respecting medical conventions in rotations and flips) to learn more structure-sensitive representations.

To address scale variations in high-resolution medical images, Dong et al. [160] construct local contrastive samples using sliding windows, enabling the model to learn consistent normal patterns across spatial locations and improving sensitivity to small lesions. PatchCL-AE [91] combines patch-level contrastive learning with autoencoder reconstruction, enhancing feature deviation in anomalous regions while preserving global reconstruction capability. To capture more complex semantic variations, Patrício et al. [161] introduce instance alignment and class-invariant constraints to the encoder, focusing on common anatomical structures while suppressing anomalous factors, and combine this with diffusion-based reconstruction differences for anomaly scoring. Tang et al. [39] incorporate contrastive constraints into a dual-decoder architecture, ensuring semantic consistency between reconstruction and attention branches, thereby amplifying feature deviations in anomalous regions. Overall, contrastive learning enables the formation of compact normal feature distributions through multi-view consistency, improving anomaly separability. However, its performance depends on the design of data augmentation and positive/negative sample construction, and inappropriate transformations may negatively affect the stability of anomaly representations.

#### 3.3.3. Summary of Self-Supervised Learning Methods

In MUAD, proxy task-based methods amplify structural deviations by introducing artificial perturbations to normal images, making them well-suited for scenarios with scarce anomaly annotations. In contrast, contrastive learning methods enforce cross-view or cross-modal consistency, leading to more compact semantic representations of normal samples and enhancing sensitivity to anomalous deviations. However, the effectiveness of these methods is highly dependent on the design of proxy tasks and augmentation strategies. If pseudo anomalies differ significantly from real lesions in morphology or semantics, generalization performance may be limited. Moreover, self-supervised representations do not directly correspond to anomaly scores, and are often combined with reconstruction-based or feature embedding-based methods.

Overall, self-supervised learning is better regarded as a representation enhancement mechanism within MUAD. From a developmental perspective, its role is gradually shifting from a feature pretraining tool to an integral component of anomaly detection frameworks. Increasing efforts incorporate anatomical priors and medical imaging knowledge into SSL task design, enabling models to learn more clinically meaningful normal patterns. Furthermore, SSL is increasingly integrated with reconstruction models and feature distribution modeling through multi-task learning, strengthening both semantic representation and anomaly deviation modeling. In the future, self-supervised learning is expected to play a more important role in medical structure-aware modeling and multi-modal knowledge integration, thereby improving semantic understanding and clinical applicability in MUAD.

### 3.4. Foundation Model-Based Methods

In recent years, the development of foundation models has driven medical unsupervised anomaly detection from traditional visual representation learning toward semantic-enhanced and general modeling paradigms. These methods are no longer limited to feature distribution modeling or reconstruction errors, but instead leverage the visual and semantic priors embedded in large-scale pre-trained models to improve anomaly detection performance. In this survey, foundation model-based studies are discussed mainly from the perspective of transferable visual-semantic priors for anomaly scoring and localization, while their specific supervision settings may vary across normal-only, zero-shot, weakly supervised, or auxiliary-data-based scenarios. According to model types and their roles, existing approaches can be categorized into vision–language model-based methods, large language model-based methods, and visual foundation model-based methods.

#### 3.4.1. Vision–Language Model-Based Methods

**Figure 14:**
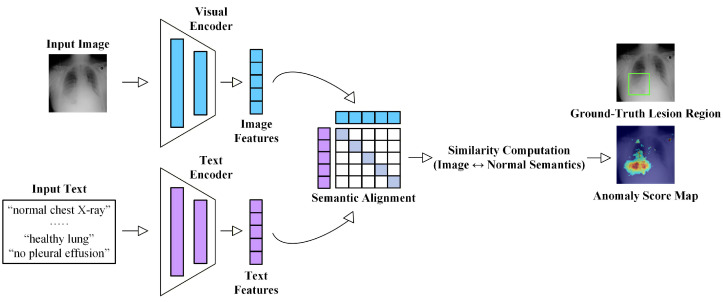
Illustration of the vision–language model framework for anomaly detection. Image features extracted by the visual encoder and text features extracted by the text encoder are aligned in a shared semantic space. The anomaly score is obtained by computing the similarity between the image representation and normal semantic descriptions.

Vision–language model (VLM)-based methods mainly utilize cross-modal pre-trained models such as CLIP [162] to map medical image features into a shared semantic space, and perform anomaly detection through image–text alignment. The general pipeline is illustrated in Figure 14.

Early studies explored the direct application of general-purpose VLMs for anomaly detection. WinCLIP [69] formulates anomaly detection as an image–text similarity problem by constructing “normal/abnormal” prompts and further employs sliding windows for local anomaly localization. FADE [163] introduces both language-guided and vision-guided mechanisms, where large language models are used to generate diverse textual prompts, and patch-level alignment modules are designed to handle anomalies at different scales. Marzullo et al. [164] conducted a systematic evaluation of CLIP-based methods on brain metastasis MRI datasets, revealing the limitations of 2D VLMs in modeling 3D medical structures.

To address the limitations of general VLMs in medical semantic understanding and spatial structure modeling, researchers have introduced medically-aware prompt learning mechanisms. These approaches construct semantic descriptions in natural language to guide VLMs toward specific medical concepts and decision boundaries. MediCLIP [93] adapts CLIP to medical scenarios through anomaly synthesis, self-supervised fine-tuning, and learnable textual prompts. Park et al. [165] design disease-related positive prompts and normal-structure-related negative prompts, and suppress abnormal activations in normal regions by leveraging differences in attention responses. Sun et al. [166] incorporate anatomical priors into prompt learning by encoding lung region information together with category semantics in the text branch, while applying region masking and image prompting in the visual branch to focus on specific anatomical regions. Lai et al. [167] further decompose prompts into perception-level and recognition-level prompts, enhancing local anomaly perception and clarifying decision boundaries in semantic space, respectively.

To improve cross-domain generalization of VLMs in medical anomaly detection, Huang et al. [92] introduce lightweight residual adapters into the visual encoder and propose pixel-level and image-level vision–language alignment losses, enabling cross-modality and cross-anatomy generalization. OFF-CLIP [99] points out that the InfoNCE loss in standard CLIP may disrupt the clustering structure of normal samples, leading to high false positive rates; it addresses this issue by introducing off-diagonal loss terms and text filtering strategies to enhance representation consistency. In few-shot scenarios, MadCLIP [100] proposes a dual-branch visual adapter to model normal and abnormal distributions separately, combined with learnable prompts and optimization objectives. AdaptCLIP [97] introduces lightweight adapters into both visual and textual encoders and employs anomaly score-driven alignment objectives to focus on anomaly-related representations rather than generic semantic consistency. WMoE-CLIP [95] further incorporates global semantic distribution sampling, wavelet-enhanced cross-modal attention, and semantic-aware mixture-of-experts prompt learning, improving the modeling of complex semantics and subtle anomalies.

More recently, VLMs have been incorporated as semantic priors in generative models. Wang et al. [168] integrate pathological VLMs into diffusion-based generation, where textual information guides reconstruction toward semantically plausible normal structures. Chen et al. [169] introduce diffusion-based denoising in CLIP feature space to refine multi-level features and improve robustness to noise and sparse anomalies. Sun et al. [170] propose a co-evolution learning framework that jointly performs anomaly detection and radiology report generation by sharing vision–language representations, establishing bidirectional constraints between localization and textual descriptions. Overall, VLM-based methods provide enhanced interpretability and high-level semantic representations for medical anomaly detection. However, their performance depends on the quality of vision–language alignment and is sensitive to prompt design and semantic representation.

#### 3.4.2. Visual Foundation Model-Based Methods

**Figure 15:**
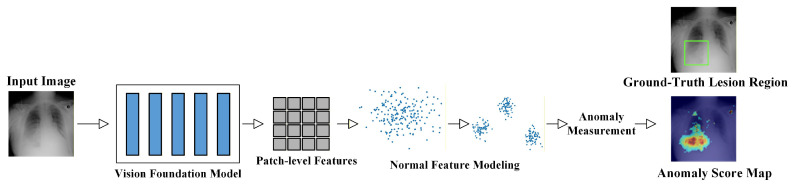
Illustration of anomaly detection using vision foundation models. Patch-level features are extracted from the input image using a vision foundation model. Anomalous regions are detected by modeling the distribution of normal features or measuring deviations from normal patterns. The example shown in the figure illustrates a clustering-based modeling approach.

Visual foundation model-based methods primarily leverage large-scale pre-trained visual encoders, such as Vision Transformer (ViT), DINO [171], and DINOv2 [172], to extract features with strong semantic representation and cross-domain generalization ability. Based on these features, anomaly detection is performed by combining feature distribution modeling, similarity measurement, or reconstruction deviation analysis. The general framework is illustrated in Figure 15. Compared with VLM-based methods, these approaches do not rely on textual prompts or cross-modal alignment, thus avoiding instability caused by prompt design or semantic mismatch. This makes them more suitable for capturing fine-grained structural anomalies in medical images that are difficult to describe in language, providing a more direct and robust purely visual modeling pathway.

Schulthess et al. [173] propose a DINOv2-based anomaly detection method that models the distribution of normal patch features using a Dirichlet process mixture model and computes anomaly scores based on similarity to cluster centers, achieving anomaly detection without requiring large-scale feature memory banks. AnomalyMoE [96] utilizes visual foundation models to extract general visual representations and employs a hierarchical mixture-of-experts architecture to model local structural anomalies, part-level semantic anomalies, and global logical anomalies. AnomalyVFM [174] introduces a three-stage synthetic anomaly generation strategy and incorporates low-rank feature adapters and lightweight decoders to efficiently adapt pre-trained visual features, transforming models such as DINOv2 into general zero-shot anomaly detection frameworks. UniADet [175] learns task weights for anomaly classification and segmentation, and decouples image-level classification and pixel-level localization across different feature levels, enabling both zero-shot and few-shot anomaly detection. VisualAD [42] introduces learnable normal and abnormal global tokens into a frozen Vision Transformer, allowing interaction with patch features through multi-layer self-attention, and performs anomaly modeling directly in the feature space, combined with spatial-aware cross-attention and feature self-alignment mechanisms.

In addition to representation-oriented visual foundation models, promptable segmentation foundation models have also attracted growing attention [176], particularly SAM [177] and its medical variants such as MedSAM [178] and SAMed [179]. MedSAM [178] adapts SAM to large-scale medical image segmentation, while SAMed [179] explores lightweight fine-tuning strategies to improve its applicability to medical images. These developments are also relevant to MUAD, as they provide a promising segmentation prior for abnormal region delineation, especially in weakly supervised or zero-shot settings. Recent anomaly-related studies have begun to explore this direction. MIAS-SAM [180] integrates the SAM encoder into a memory-bank-based anomaly detection pipeline to compute anomaly maps, and then automatically converts the anomaly map into a point prompt for the SAM decoder, refining anomaly localization into threshold-free segmentation. Yang et al. [181] further show that SAM can be adapted for anomaly segmentation through task-specific tuning. Their self-perception tuning strategy enhances SAM’s perception of abnormal regions under domain shift and improves prompt-robust anomaly localization. A more direct integration is explored in STLM [182], which uses a fixed SAM as a teacher to distill generalized knowledge into a lightweight two-stream anomaly detection model and generates anomaly maps from the discrepancy between the two streams.

These studies suggest that SAM-like models may provide a useful complementary pathway for MUAD, especially in anomaly localization, mask refinement, and teacher-guided anomaly modeling. However, their role in MUAD is still at an early exploratory stage. Future research is needed to better understand how promptable segmentation foundation models can be more effectively integrated with anomaly scoring, medical semantics, and complex 3D clinical scenarios.

Overall, visual foundation model-based methods effectively leverage the general visual representations learned from large-scale pretraining, enabling anomaly detection with strong generalization ability without relying on language priors. Recent SAM-based developments further enrich this purely visual route by offering an additional option for localization refinement and task-specific adaptation.

#### 3.4.3. Large Language Model-Based Methods

**Figure 16:**
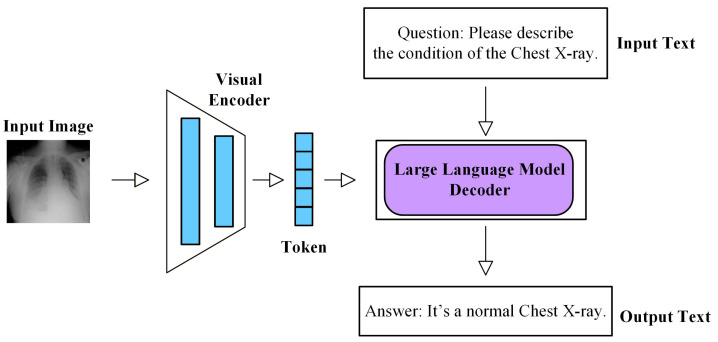
Illustration of anomaly detection assisted by large language models. Medical images are first processed by a visual encoder to obtain visual tokens, which are combined with textual prompts and fed into a large language model. The model performs semantic reasoning to generate diagnostic descriptions or anomaly assessment results.

Different from vision–language models that emphasize cross-modal alignment, large language models (LLMs) in medical anomaly detection primarily serve as semantic reasoning and knowledge enhancement components. These methods organize anomaly cues into semantically interpretable information and leverage mechanisms such as prompt generation, instruction understanding, and language-based explanation to enhance open-set recognition capability and clinical interpretability. The general pipeline is illustrated in Figure 16.

Early explorations employ LLMs as prompt generators to expand semantic coverage and diversity of anomaly descriptions. For instance, FADE [163] utilizes LLMs to automatically generate large-scale and diverse textual prompts, enabling more comprehensive descriptions of potential anomalies, and effectively compensating for the limitations of visual models in semantic modeling. With further development, LLMs have gradually evolved from auxiliary generation tools to components that participate in anomaly reasoning and cross-modal alignment. As illustrated in Figure 16, medical images are first encoded into visual tokens, which are then jointly processed with user queries or textual prompts by LLMs for unified reasoning. This allows the model to combine visual information with medical knowledge to generate diagnostic descriptions or anomaly predictions, thereby enabling semantic analysis and interpretation of abnormal states. Building upon this paradigm, Li et al. [183] introduce a query-driven cross-modal attention mechanism, where textual queries actively guide the extraction of anomaly-related visual representations, enhancing alignment between visual features and semantic queries. Xu et al. [101] address the limitations of multi-modal medical models in fine-grained anomaly description and causal reasoning by constructing instruction datasets and evaluation benchmarks for anomaly detection and reasoning, and propose a “re-inspection” feature matching mechanism to refine attention on suspicious regions. Furthermore, Zhou et al. [184] propose a two-stage training strategy, including anomaly-aware instruction tuning and anomaly-aware reward learning. By introducing anomaly-related reward signals, the model is encouraged to focus on abnormal regions during report generation and produce explanations with localization evidence, thereby improving cross-modal generalization. Overall, LLM-based methods in MUAD are evolving from prompt expansion and textual explanation toward anomaly-aware instruction learning and semantic reasoning frameworks. Their primary role is to complement medical semantic priors and enhance the interpretability of anomaly detection results.

#### 3.4.4. Summary of Foundation Model-Based Methods

Foundation model-based methods significantly expand the modeling scope of medical unsupervised anomaly detection by leveraging visual and semantic priors learned from large-scale pretraining. Vision–language models construct semantic reference spaces for anomaly discrimination through cross-modal alignment, while visual foundation models enable robust anomaly modeling within purely visual feature spaces by exploiting strong visual representations. In this context, recent SAM-based adaptations also suggest a potential direction for enhancing anomaly localization. Large language models further enhance interpretability and clinical readability through semantic reasoning and knowledge augmentation. Overall, foundation models have driven MUAD from traditional visual statistical modeling toward a unified framework that integrates visual representation, semantic priors, and reasoning capabilities.

However, most existing methods still rely on visual feature differences or distribution deviations as the primary anomaly metrics, while language models often function as auxiliary components for semantic enrichment and explanation rather than directly participating in low-level anomaly discrimination. In addition, their performance is influenced by the quality of cross-modal alignment and prompt design strategies. In high-resolution medical imaging scenarios, challenges such as computational complexity and limited capability for fine-grained localization remain. Future research should focus on deeper integration of visual representations, medical semantic knowledge, and anomaly decision mechanisms, in order to further improve the generalization ability and interpretability of MUAD in complex clinical environments.

## 4. Evaluation Metrics and Benchmark Datasets

### 4.1. Evaluation Metrics

The evaluation of medical unsupervised anomaly detection (MUAD) is typically conducted at two levels according to the detection granularity: image-level and pixel-level, with different tasks emphasizing different evaluation criteria. For image-level anomaly detection, the model outputs a global anomaly score to determine whether an entire image is abnormal. The most commonly used metrics include the Area Under the Receiver Operating Characteristic Curve (AUROC) and the Area Under the Precision–Recall Curve (AUPRC). Among them, AUROC is relatively insensitive to class imbalance, whereas AUPRC better reflects the actual detection capability in medical scenarios where anomalous samples are extremely scarce. In addition to these metrics, some studies also report Accuracy (ACC), Precision (PRE), Sensitivity/Recall (SEN), Specificity (SPE), and F1-score to provide a more intuitive characterization of the trade-off between missed detections and false positives, which is also aligned with practical clinical screening requirements. Nevertheless, these image-level metrics mainly reflect the overall separability between normal and abnormal samples. In MUAD, where lesions may be small, sparse, or only partially detected, AUROC and AUPRC may still remain relatively high even when the spatial localization is inaccurate or clinically important abnormalities are incompletely captured.

For pixel-level anomaly localization tasks (as illustrated in Figure 3), the model is required to produce spatial anomaly maps. Common evaluation metrics include pixel-level AUROC, Dice Similarity Coefficient (Dice), Intersection over Union (IoU), and the Area Under the Per-Region Overlap curve (AUPRO). These metrics not only assess whether anomalies are detected but also emphasize spatial consistency and localization accuracy, making them more suitable for lesion localization and quantitative analysis. However, Dice and IoU are still strongly influenced by lesion size and overall overlap, and may not adequately reflect boundary deviation, spatial misalignment, or the clinical importance of missed small lesions. In particular, for cases with multiple lesions or highly imbalanced lesion sizes, global overlap-based metrics may favor methods that capture large lesions while underestimating failures on smaller but clinically relevant abnormalities.

To address these limitations, recent studies have begun to explore more fine-grained or clinically meaningful evaluation criteria. For example, the BraTS 2023 official evaluation introduced lesion-wise Dice and lesion-wise HD95 to assess performance at the lesion level rather than only at the whole-image level, with the explicit aim of reducing the bias of traditional global metrics toward large lesions and better reflecting how well a model detects and segments multiple individual lesions [21]. In addition, for reconstruction- and generation-based MUAD methods, recent work has proposed normative-learning-oriented metrics, including the Restoration Quality Index (RQI), the Anomaly to Healthy Index (AHI), and the Healthy Conservation and Anomaly Correction Index (CACI), in order to evaluate not only anomaly detection itself but also the quality of pseudo-healthy restoration and the fidelity of learned normal representations [3]. These metrics highlight that, for generative MUAD models, evaluation should go beyond anomaly maps alone and also consider whether healthy structures are preserved and pathological regions are plausibly corrected.

Overall, these metrics remain the mainstream evaluation tools in MUAD and still provide an important basis for comparison. At the same time, given the complexity of medical anomalies, future evaluation protocols may benefit from incorporating lesion-level assessment, calibration of anomaly scores, uncertainty-aware evaluation, and other clinically meaningful criteria that better reflect lesion detectability, localization reliability, and potential diagnostic relevance.

### 4.2. Common Datasets and Experimental Benchmarks

Due to the complexity of medical imaging sources, the diversity of imaging modalities, and variations in annotation protocols, MUAD research has not yet established a unified evaluation benchmark in terms of experimental settings and dataset selection. Existing studies often construct experimental setups tailored to specific modalities or clinical scenarios, such as chest X-ray, brain MRI, chest CT, or retinal OCT. This diversity makes it difficult to perform direct and fair comparisons of model performance across different studies.

Therefore, instead of simply aggregating reported performance metrics, it is more meaningful to systematically review commonly used datasets in order to better understand the experimental contexts and applicability of different methods.

From the current literature, commonly used MUAD datasets mainly cover multiple medical imaging modalities, including chest X-ray, brain MRI, chest CT, histopathology images, and retinal OCT. These datasets differ significantly in terms of anomaly types, annotation granularity, and task settings. For example, some datasets only provide image-level labels for anomaly screening, while others offer pixel-level or region-level annotations for anomaly localization and segmentation tasks. To provide a clearer overview of data resources in MUAD research, Table 3 summarizes representative medical anomaly detection datasets in recent years, including their imaging modality, anomaly categories, annotation granularity, and typical application scenarios.

Beyond modality and annotation differences, domain shift across datasets is another critical issue in MUAD evaluation. Even within the same modality, medical datasets collected from different institutions may vary substantially in imaging devices, acquisition protocols, reconstruction pipelines, patient populations, and disease composition. These factors can change image contrast, noise characteristics, spatial resolution, and even the apparent distribution of normal anatomy, thereby affecting the stability of anomaly modeling and making models trained on one dataset difficult to generalize reliably to another. As a result, the heterogeneity of datasets is not only a matter of benchmark diversity, but also an important source of cross-dataset generalization difficulty. The broader implications of this issue for robustness and clinical deployment are further discussed in Section 5.4.

## 5. Comprehensive Discussion and Future Directions

### 5.1. Method-Level Challenges: A Comprehensive Review and Analysis

From a methodological perspective, existing MUAD approaches all follow the general paradigm of learning normality and detecting deviations, yet they differ substantially in modeling assumptions, anomaly evidence, and clinical applicability. Reconstruction-based methods provide intuitive anomaly maps, but they are vulnerable to anomaly leakage and often struggle with subtle semantic abnormalities. Feature embedding-based methods generally offer better stability and stronger distribution modeling, but their performance is still constrained by the semantic gap between natural-image pretraining and medical imaging. Self-supervised learning methods improve representation quality by introducing proxy tasks or contrastive constraints, although their effectiveness depends heavily on the realism of pseudo anomalies and the design of augmentation strategies. Foundation model-based approaches bring stronger semantic priors and improved interpretability, but their direct contribution to fine-grained anomaly quantification and localization remains limited.

To facilitate a more explicit cross-method comparison, Table 4 summarizes major MUAD method categories in terms of their core mechanisms, strengths, limitations, suitable anomaly types, and computational complexity. Moreover, in current MUAD research, interpretability is still mainly conveyed through anomaly maps or score visualizations. Although these outputs can provide intuitive spatial cues, they often remain indirect evidence, and more rigorous validation of whether such explanations are faithful to model behavior and clinically meaningful is still relatively limited.

As shown in Table 4, different MUAD paradigms exhibit clear trade-offs in anomaly modeling ability, localization performance, interpretability, and computational cost. Reconstruction-based methods are intuitive and naturally suitable for anomaly localization, especially for structural abnormalities with relatively clear morphological deviations, but they remain limited by anomaly leakage and insufficient semantic discrimination. In contrast, feature embedding-based methods are generally more stable and better suited for modeling normal distributions in deep feature spaces, making them effective for a wider range of structural and distributional anomalies, although they often rely heavily on the quality of pretrained representations. Self-supervised learning-based methods are better regarded as representation enhancement strategies, as their gains mainly come from improving the compactness and discriminability of normal features, but their robustness is sensitive to proxy-task design and augmentation realism. Foundation model-based methods further extend MUAD toward high-level semantic reasoning and cross-domain generalization, yet their advantages are currently more evident in interpretability and open-set understanding than in precise low-level anomaly quantification.

Overall, no single methodological family is uniformly superior; instead, different paradigms are complementary and should be selected or combined according to anomaly type, imaging modality, and clinical requirements.

From a broader perspective, existing unsupervised methods often simplify anomalies as a single form of “deviation,” while overlooking the multi-level inconsistencies of medical anomalies across structural, semantic, and functional dimensions. Future research should move beyond optimizing a single anomaly score and instead focus on jointly modeling multi-level inconsistencies, such as structural inconsistency, semantic inconsistency, and cross-modal inconsistency, to improve both robustness and interpretability. Furthermore, “unsupervised” does not imply the absence of prior knowledge. Incorporating anatomical constraints, medical knowledge priors, or uncertainty modeling mechanisms under limited supervision is expected to be a key direction for advancing MUAD.

### 5.2. Model-Level Trends and Evolution Directions

From a modeling perspective, MUAD has evolved from early single-strategy designs toward multi-mechanism collaborative modeling. This evolution is driven by the intrinsic complexity of medical anomalies in terms of structural scale, semantic hierarchy, and spatial distribution, which makes it difficult for a single representation or decision pathway to robustly capture abnormal patterns. Recent model designs exhibit several notable trends:

1. **Multi-scale structural modeling has become a fundamental component.** Different scales encode different levels of structural and semantic information in medical images. By integrating multi-scale representations, models can simultaneously capture global anatomical consistency and local lesion abnormalities, thereby alleviating instability caused by scale variations. In recent studies, multi-scale feature extraction, cross-level interaction, and multi-resolution consistency constraints have been widely adopted. For instance, Tian et al. [106] incorporate cross-level attention within an autoencoder framework, while pDDPM [121] and mDDPM [82] enhance local–global modeling via patch-based or masked diffusion. Methods such as PaDiM [58], SPADE [138], and CFLOW [79] also rely on multi-scale feature spaces for modeling normal distributions. These observations indicate that multi-scale modeling has evolved from a performance enhancement technique into a fundamental design principle for complex medical anomaly detection.
2. **Transition from single-path anomaly detection to multi-view collaborative modeling.** Multi-branch or multi-view frameworks characterize anomalies from different perspectives, such as reconstruction consistency, feature distribution stability, and semantic similarity, enabling better modeling of multi-faceted inconsistencies in medical anomalies. Recent works increasingly emphasize the complementarity among different mechanisms. For example, DRÆM [151] models anomalies via joint reconstruction and discrimination branches; Zhao et al. [38] combine normalizing flow with reconstruction frameworks; FADE [163], MadCLIP [100], and AnomalyMoE [96] explore multi-view modeling through visual guidance, normal/abnormal distribution separation, and multi-expert collaboration, respectively. However, such designs also introduce trade-offs between model complexity and training stability.
3. **“Frozen backbone + lightweight adaptation” has become a mainstream paradigm.** Fully end-to-end training may disrupt the general structural representations learned during large-scale pretraining, whereas lightweight adaptation modules allow models to retain stable representations while focusing on anomaly-relevant subspaces. For example, Tang et al. [83] employ a stop-gradient strategy to mitigate the semantic gap between natural and medical images; Huang et al. [92] and AdaptCLIP [97] introduce lightweight adapters into vision–language models; VisualAD [42] incorporates learnable tokens into frozen Vision Transformers to model normal and abnormal patterns. This paradigm serves as an effective bridge between general-purpose pretrained representations and domain-specific anomaly detection tasks.
4. **Increasing emphasis on anomaly score stability and uncertainty modeling.** Recent studies have shifted from purely improving detection accuracy toward enhancing the reliability and consistency of anomaly predictions, which is critical in medical applications. Techniques such as probabilistic modeling, ensemble strategies, and uncertainty estimation are increasingly adopted. For instance, Huang et al. [46] introduce pixel-level Bayesian uncertainty into prototype learning to model ambiguous boundaries; DDAD-ASR [84] refines anomaly scores via a self-supervised refinement module; OFF-CLIP [99] improves clustering consistency of normal samples to reduce false positives. These developments suggest that future MUAD models will place greater emphasis on probabilistic reasoning, uncertainty estimation, and multi-source consistency validation.

In summary, model evolution is driving MUAD from single-path mapping toward structure-aware, multi-view, and stability-oriented composite models. However, increased model complexity does not necessarily guarantee improved anomaly discrimination. Achieving a balance among representation capacity, reliability of anomaly quantification, and computational efficiency remains a central challenge for future model design.

### 5.3. Task-Level Expansion and Multi-Task Integration Trends

At the task level, MUAD is evolving from primarily image-level anomaly classification toward pixel-level anomaly localization. While a single image-level anomaly score is useful for screening abnormal samples, it is insufficient to meet clinical requirements regarding lesion location, extent, and morphology. Therefore, pixel-level anomaly localization has gradually become an important criterion for evaluating model effectiveness. Meanwhile, anomaly detection is increasingly integrated with related tasks such as segmentation, classification, image reconstruction, and report generation. Through multi-task joint modeling, models can better learn both the spatial boundaries and semantic attributes of abnormal regions, thereby extending the applicability of anomaly detection across different organs, disease types, and clinical scenarios. For example, Sun et al. [170] combine anomaly detection with medical report generation, enabling the model to produce diagnostic descriptions while detecting abnormalities.Such multi-task learning frameworks introduce complementary supervisory signals, allowing models to characterize anomalies from multiple perspectives. This not only improves robustness and generalization but also promotes the evolution of MUAD from a single-task detection paradigm toward a more comprehensive intelligent diagnostic module.

Besides, a more specific and underexplored direction is to connect MUAD with downstream clinical tasks such as prognosis assessment, longitudinal follow-up, and treatment response analysis [190,191]. In real clinical practice, detecting whether an image is abnormal is often only the first step; clinicians are also concerned with how abnormal findings evolve over time, whether they imply higher risk, and how they should be described in a clinically meaningful way. From this perspective, future MUAD systems may gradually evolve from standalone detection modules into more comprehensive task interfaces that support lesion localization, semantic description, and subsequent clinical decision-making.

### 5.4. Technology Integration Trends and Future Research Directions

As MUAD methods continue to evolve, future research should not only pursue higher detection performance, but also strengthen the connection between methodological advances and clinical use. Although most existing MUAD systems are still evaluated mainly in research settings, recent studies suggest that anomaly detection is particularly relevant to practical scenarios such as rare abnormality discovery, health screening, and open-set medical analysis, while its translation to real clinical workflows remains constrained by generalization, interpretability, evaluation, and deployment issues [4]. From this perspective, several key directions deserve further attention:

**(1) Clinical consistency and calibration of anomaly scores.** Clinical relevance and clinically usable anomaly quantification. MUAD has clear potential clinical value, particularly in supporting large-scale screening, suspicious case prioritization, candidate lesion prompting, and open-set abnormality discovery under limited-annotation conditions. Recent benchmark-oriented studies have also emphasized the importance of anomaly detection for rare disease recognition and health screening in medical imaging [11,192]. However, current anomaly scores are usually derived from reconstruction errors, feature deviations, or similarity discrepancies, and are therefore not naturally aligned with clinically meaningful factors such as lesion type, severity, progression, or diagnostic actionability. This mismatch makes it difficult to directly translate model outputs into practical clinical decisions. Future work should thus focus on clinically usable anomaly quantification frameworks by incorporating uncertainty estimation, score calibration, semantic grounding, and workflow-oriented presentation, so that anomaly predictions can better reflect not only the presence of abnormalities but also their confidence and clinical relevance. For example, calibrated anomaly scores may be used to prioritize suspicious cases according to risk level, while lesion-aware anomaly quantification may provide more actionable cues for follow-up review and clinical reporting.

**(2) Generalization and robustness across institutions and imaging conditions.** Generalization across datasets remains a major challenge for MUAD, especially across modalities, organs, and clinical scenarios. Recent studies suggest that foundation model-based methods show relatively better transferability, indicating that stronger pretrained representations may provide a promising direction for improving cross-dataset robustness [42,93,101]. Moreover, domain shift is not limited to cross-modality settings. Even within the same modality, differences in imaging devices, acquisition protocols, reconstruction pipelines, and patient populations can substantially affect image statistics and normal anatomical distributions, thereby reducing the stability of anomaly scoring in unseen environments. This makes cross-site and cross-institution robustness a critical issue for clinical deployment [193]. Recent MUAD studies have further emphasized that clinically useful systems must remain robust across scanning protocols and patient demographics, rather than performing well only under controlled experimental conditions [194,195]. Future research may therefore benefit from foundation model adaptation, federated learning for privacy-preserving multi-center collaboration, and domain generalization, few-shot adaptation, or zero-shot transfer techniques to enhance robustness under realistic clinical variability.

**(3) Scalable modeling for high-resolution, 3D, and workflow-oriented scenarios.** Medical data such as 3D CT, 3D MRI, and whole slide imaging (WSI) exhibit extremely high resolution and complex spatial structures. Directly applying 2D models often leads to excessive computational costs and loss of inter-slice or inter-region consistency. Future work should focus on scalable modeling paradigms, including patch-based processing, where recent studies have already explored patch-wise or masked diffusion for efficient local modeling [82,121], sliding-window strategies, as exemplified by sliding-window-based self-supervised learning for high-resolution images [160], sparse attention, as well as coarse-to-fine inference and multi-stage candidate filtering. Beyond computational efficiency, such paradigms may also support more practical MUAD settings, including volumetric lesion continuity analysis, large-scale WSI screening, and multi-stage candidate filtering for downstream clinical workflows.

**(4) Integration of medical priors and external knowledge.** Purely data-driven unsupervised learning may capture spurious correlations that are inconsistent with medical knowledge. A promising direction is to incorporate anatomical constraints, symmetry priors, inter-organ spatial relationships, and medical knowledge graphs into the learning process in a controlled manner. For instance, anatomical segmentation models, organ templates, topological constraints, or radiomic-guided priors can enhance normal structure modeling [196], as already suggested by structure-guided modeling with tissue segmentation maps [81] and anatomy-aware proxy-task design [152]. Likewise, radiology reports, large language models, or retrieval-augmented frameworks can introduce higher-level semantic knowledge, as reflected in recent work on EHR-guided diffusion reconstruction [28] and anomaly-aware report generation or reasoning with vision–language and language models [170,184]. The key challenge lies in achieving effective synergy between prior knowledge and data-driven representations while avoiding the introduction of additional biases. In the future, such integration may help MUAD move from merely detecting abnormality toward describing, contextualizing, and reasoning about abnormal findings in a clinically meaningful way.

**(5) Toward rigorous evaluation of interpretability.** In current MUAD research, anomaly heatmaps, anomaly score maps, and pixel-level predictions are often taken as interpretable outputs, yet they generally serve only as indirect evidence and cannot by themselves constitute explanations. More rigorous evaluation protocols are therefore needed. Future studies may assess interpretability from at least three complementary perspectives. First, clinician-centered studies can examine whether explanations help clinicians understand model outputs, improve trust, or support decision-making in realistic reading settings. Recent work in medical AI suggests that such user studies are important, because explanations do not automatically lead to better reliance or clinical utility [197,198]. Second, faithfulness-based evaluation can test whether highlighted regions are truly related to model predictions, for example through perturbation-based analysis or other fidelity metrics [199]. Third, counterfactual- or pseudo-healthy-based assessment is particularly relevant for generative MUAD methods, where explanation quality may also be reflected by the plausibility of restored images and the preservation of healthy structures. Recent generative anomaly detection studies have already proposed metrics such as RQI, AHI, and CACI in this direction [3,200]. Together, these perspectives may help make interpretability evaluation in MUAD more systematic and clinically meaningful.

**(6) Toward universal foundation models and standardized benchmarks.** Building general-purpose anomaly detection models for multi-modal medical data requires not only stronger foundation models, but also more clinically meaningful evaluation settings. Recent comparative benchmark studies have pointed out that the field still lacks sufficiently fair and comprehensive evaluation, and that conclusions drawn from simplified public datasets may not fully reflect performance in heterogeneous real-world scenarios [192,201]. Future efforts may therefore focus on leveraging large-scale foundation models to enable cross-organ, cross-modality, and cross-task anomaly detection, while at the same time promoting standardized benchmarks across institutions, organs, modalities, and tasks, including unified data splits, annotation granularity, and evaluation metrics. Additional evaluation dimensions, such as calibration, robustness, external validation, and clinical usability, should also be incorporated to support fairer and more reliable comparisons. Because MUAD is well suited to rare, open-set, and previously unseen abnormalities, future foundation-model-based systems may support not only anomaly detection, but also early screening, case triage, report generation, longitudinal monitoring, and potentially prognosis-related analysis.

**(7) Post-deployment monitoring and governance.** Even if MUAD systems achieve satisfactory performance in validation settings, safe clinical use still requires continuous monitoring after deployment [202]. Future research and application efforts should therefore also consider post-deployment issues such as performance drift, safety reporting, human–AI interaction, and governance mechanisms, so that anomaly detection systems can remain reliable, transparent, and clinically accountable in routine practice. For example, deployed systems may need routine drift monitoring across sites, devices, or patient populations, as well as human review when anomaly predictions become unstable in out-of-distribution cases.

## 6. Conclusions

This paper provides a comprehensive review of recent advances in medical unsupervised anomaly detection. We systematically summarize major methodological paradigms, including image reconstruction-based methods, feature embedding-based methods, self-supervised learning approaches, and foundation model-based techniques, highlighting their core principles, technical evolution, and application scenarios. Overall, MUAD has evolved from early approaches relying on low-level visual discrepancies toward a multi-paradigm framework that integrates multi-scale structural modeling and high-level semantic representation. However, due to the inherent complexity of medical images, the diversity of anomaly semantics, and the constraints of the unsupervised setting, existing methods still face challenges in anomaly quantification reliability, cross-institution generalization, and clinical interpretability. Looking forward, the integration of multi-task learning, medical prior knowledge, multi-modal information, and foundation models is expected to further advance MUAD. Beyond methodological progress, MUAD also shows practical potential in real-world scenarios such as large-scale pre-screening, suspicious case triage, rare abnormality discovery, and clinical workflow support under limited-annotation conditions. Nevertheless, translating current MUAD systems from benchmark evaluation to routine clinical use still requires further advances in robustness, calibration, interpretability, external validation, and post-deployment monitoring. These developments will enable more robust, interpretable, and clinically applicable anomaly detection systems, ultimately facilitating their deployment in real-world medical screening and computer-aided diagnosis.

## Figures and Tables

**Table 1:** Comparison between industrial imaging unsupervised anomaly detection (IUAD) and medical imaging unsupervised anomaly detection (MUAD).

Task Domain	MUAD	IUAD
**Task Objective**	Detect clinically meaningful deviations from normal anatomy or physiology for screening, diagnosis, or lesion localization.	Detect structural or surface defects for quality inspection and process control.
**Modality Characteristics**	Multi-modal data (e.g., MRI, CT, X-ray, ultrasound, endoscopy, WSI), often including 2D and 3D images.	Mostly 2D images acquired under controlled conditions, commonly using RGB or industrial cameras.
**Visual Characteristics**	Complex anatomical structures, strong inter-subject variability, frequent noise/artifacts, and multi-scale patterns.	Relatively regular structures, simple backgrounds, limited intra-class variation, and repetitive textures.
**Anomaly Characteristics**	Lesions often have ambiguous boundaries, semantic complexity, and large variation in size, shape, and location.	Defects are usually localized, visually explicit, and with relatively clear boundaries.
**Annotation and Data Constraints**	Abnormal samples are scarce; annotations require clinical expertise and are constrained by privacy and ethics.	Annotation is relatively easier, and defect data can often be collected or synthesized more conveniently.
**Modeling Priorities**	Semantic structure modeling, modality adaptation, interpretability, and clinical reliability.	Texture sensitivity, efficiency, stability, and real-time deployment.

**Table 3:** Overview of 16 commonly used public medical image datasets in anomaly detection research, including imaging modality, dataset split, task type, annotation type, anomaly categories, and typical application scenarios. The dataset splits shown here follow representative studies in the literature and may vary across different works.

Dataset	Modality	Split	Task Type	Annotation	Anomaly Type	Description	URL
RSNA	X-ray	Train: 3851 normal images; Test: 1000 normal + 1000 abnormal images [20]	Image-level	Image-level labels	Lung opacity (pneumonia-related lesions)	Chest X-ray dataset released for the RSNA Pneumonia Detection Challenge, containing 26,684 DICOM images annotated by radiologists into categories such as Normal, Lung Opacity, and No Lung Opacity/Not Normal. It is widely used in chest anomaly detection research.	https://www.kaggle.com/c/rsna-pneumonia-detection-challenge (accessed on 28 April 2026)
VinDr-CXR [16]	X-ray	Train: 4000 normal images; Test: 1000 normal + 1000 abnormal images [20]	Image-level	Bounding boxes + image-level labels	Cardiomegaly, atelectasis, consolidation, nodule/mass, pleural effusion, pneumothorax, and other thoracic abnormalities	A high-quality chest X-ray dataset collected from multiple major hospitals in Vietnam, containing about 15,000 images with 14 abnormality categories annotated by radiologists using lesion-level bounding boxes and global diagnostic labels.	https://vindr.ai/cxr (accessed on 28 April 2026)
Stanford CheXpert [17]	X-ray	Train: 5249 normal + 23,671 abnormal images; Test: 250 normal + 250 abnormal images [19,90]	Image-level	Image-level labels	Cardiomegaly, atelectasis, consolidation, edema, pleural effusion, pneumothorax, pneumonia, and other thoracic diseases	A large-scale chest X-ray dataset released by Stanford University, containing about 224,000 images with 14 thoracic disease labels and uncertainty annotations. It is one of the most widely used benchmarks in medical image analysis and anomaly detection.	https://stanfordmlgroup.github.io/competitions/chexpert/ (accessed on 28 April 2026)
COVID-19 [185]	X-ray	Train: 29,187 images (8085 normal, 5555 non-COVID pneumonia, 15,547 COVID pneumonia); Val: 400 images; Test: 400 images [19]	Image-level	Image-level labels	COVID-19 pneumonia, non-COVID pneumonia, and other pulmonary infection abnormalities	A public chest X-ray dataset for COVID-19 detection research, containing normal, non-COVID pneumonia, and COVID pneumonia categories, commonly used for pulmonary infection anomaly detection.	https://github.com/lindawangg/COVID-Net (accessed on 28 April 2026)
Brain MRI [21,186,187]	MRI	Train: 3743 normal slices; Test: 416 normal slices + 1000 abnormal slices [125]	Image-level/ Pixel-level	Pixel-level tumor masks	Brain tumor-related abnormalities (enhancing tumor, edema, necrotic core)	BraTS-based brain MRI benchmark widely used for brain tumor detection and localization, providing multi-modal MRI scans with pixel-level tumor annotations.	https://www.med.upenn.edu/cbica/brats/ (accessed on 28 April 2026)
Brain Tumor MRI	MRI	Train: 1000 normal images; Test: 600 normal + 600 abnormal images	Image-level	Image-level labels	Brain tumors (e.g., glioma, meningioma)	A public brain MRI dataset containing normal images and multiple tumor categories, commonly used for brain tumor detection and classification.	https://www.kaggle.com/datasets/masoudnickparvar/brain-tumor-mri-dataset (accessed on 28 April 2026)
IXI	MRI	Train: 358 scans; Val: 44 scans; Test: 158 scans [82,121]	Pixel-level	No anomaly labels (healthy subjects only)	None (healthy brain structures)	A public healthy brain MRI dataset collected from multiple centers, including T1/T2 scans. It is commonly used as normal training data, while anomaly detection is evaluated on tumor datasets such as BraTS.	https://brain-development.org/ixi-dataset/ (accessed on 28 April 2026)
BraTS2021 [21]	MRI	Train: 4211 normal slices; Test: 828 normal slices + 1948 tumor slices [11]	Pixel-level	Pixel-level tumor masks	Brain tumors (enhancing tumor, edema, necrotic core)	A version of the BraTS brain tumor challenge dataset, providing multi-modal MRI with voxel-level tumor annotations. In anomaly detection, the FLAIR modality is often converted into 2D slices for pixel-level localization tasks.	https://www.med.upenn.edu/cbica/brats2021/ (accessed on 28 April 2026)
LiTS [188]	CT	Train: 5820 normal slices; Test: 647 normal slices + 1000 tumor slices [125]	Image-level/ Pixel-level	Pixel-level lesion masks	Liver tumor lesions	A liver tumor segmentation challenge dataset containing 130 abdominal CT volumes with liver and tumor annotations. In anomaly detection, tumor regions are usually regarded as anomalies and 2D slices are extracted from 3D volumes for training and evaluation.	https://competitions.codalab.org/competitions/17094 (accessed on 28 April 2026)
HeadCT	CT	Test: 100 normal images + 100 abnormal images [174,175]	Image-level	Image-level labels	Intracranial hemorrhage	A public head CT dataset containing normal scans and hemorrhage cases. Due to its relatively small scale, it is often used to evaluate cross-dataset generalization in anomaly detection.	https://www.kaggle.com/datasets/felipekitamura/head-ct-hemorrhage (accessed on 28 April 2026)
LAG [31]	Retinal Fundus	Train: 1500 normal images; Test: 811 normal + 811 abnormal images	Image-level	Image-level labels	Glaucoma (optic disc–cup structural abnormality)	The LAG dataset contains fundus photographs collected from Beijing Tongren Hospital, including normal retinal images and glaucoma cases. It is widely used for fine-grained retinal anomaly detection.	https://github.com/smilell/AG-CNN (accessed on 28 April 2026)
ADAM [189]	Retinal Fundus	Train: 222 normal images; Test: 89 normal + 89 abnormal images [32]	Image-level/ Pixel-level	Pixel-/region-level lesion annotations	AMD-related lesions (drusen, exudate, hemorrhage, scar, etc.)	A dataset for age-related macular degeneration (AMD), providing high-resolution fundus images with lesion annotations and multiple retinal lesion types. It is commonly used for both image-level classification and pixel-level anomaly localization.	https://amd.grand-challenge.org/ADAM-Finals/ (accessed on 28 April 2026)
OCT2017 [34]	OCT	Train: 26,315 normal images; Test: 250 normal + 750 abnormal images [35]	Image-level	Image-level labels	Choroidal neovascularization (CNV), diabetic macular edema (DME), drusen	A retinal OCT dataset acquired using Spectralis OCT devices, containing normal samples and multiple retinal disease categories, widely used for retinal anomaly detection and disease classification.	https://data.mendeley.com/datasets/rscbjbr9sj (accessed on 28 April 2026)
ISIC2018 [37]	Dermoscopy	Train: 6705 normal images (NV); Test: 909 normal + 603 abnormal images [11]	Image-level	Image-level labels	Melanoma, basal cell carcinoma, benign keratosis, and other skin lesions	A skin lesion challenge dataset released by the International Skin Imaging Collaboration (ISIC). In anomaly detection studies, nevus (NV) is usually treated as the normal class, while the remaining lesion categories are treated as anomalies.	https://challenge.isic-archive.com (accessed on 28 April 2026)
Hyper-Kvasir [45]	Endoscopy	Train: 1600 normal images; Test: 500 normal + 1000 abnormal images [47]	Image-level/ Pixel-level	Image-level labels + segmentation masks	Colorectal polyps	A large-scale gastrointestinal endoscopy dataset containing multiple digestive structures and lesion categories. In anomaly detection, healthy endoscopic images are usually used as normal samples, while polyp images with segmentation masks are used for detection and localization evaluation.	https://datasets.simula.no/hyper-kvasir/ (accessed on 28 April 2026)
Camelyon16 [48]	WSI	Train: 5088 normal patches; Test: 1120 normal + 1113 abnormal patches [11]	Image-level/ Pixel-level	Patch-/region-level annotations	Lymph node metastatic cancer cells	A breast cancer lymph node metastasis detection challenge dataset containing high-resolution whole slide images (WSIs). In anomaly detection studies, WSIs are typically divided into small patches for training and evaluation.	https://camelyon16.grand-challenge.org (accessed on 28 April 2026)

**Table 4:** Comparison of major MUAD method categories in terms of core mechanisms, strengths, limitations, suitable anomaly types, and computational complexity.

Category	Method	Core Idea	Advantages	Limitations	Suitable Anomaly Types	Complexity
Image Reconstruction	AE-based methods	Learn to reconstruct normal images and characterize anomalies through reconstruction errors.	(1) Simple architecture and stable training. (2) Can generate anomaly maps in an intuitive manner. (3) Relatively suitable for modalities with stable anatomical priors.	(1) Prone to identity mapping and anomaly leakage. (2) Insufficient recovery of high-frequency details. (3) Limited sensitivity to complex semantic anomalies and subtle lesions.	Suitable for relatively obvious structural abnormalities, such as brain space-occupying lesions in MRI, layered structural disruption in OCT, and local organ morphological abnormalities in CT.	Moderate
Image Reconstruction	VAE-based methods	Perform probabilistic modeling of the latent distribution of normal samples and reconstruct them.	(1) Latent space is continuous and can be regularized. (2) Can jointly use reconstruction errors and distribution deviations. (3) Theoretically more suitable for normal distribution modeling.	(1) KL regularization and pixel-wise losses may lead to blurry reconstructions. (2) Boundaries are often unclear. (3) Limited ability to distinguish fine-grained texture anomalies and subtle lesions.	Suitable for MRI brain tumors, demyelinating lesions, and CT abnormalities with relatively obvious distribution shifts.	Moderate
Image Reconstruction	GAN-based methods	Learn the distribution of normal images through adversarial generation and reconstruct pseudo-normal images.	(1) Sharper texture and boundary reconstruction. (2) More suitable for pseudo-normal generation. (3) Helpful for enhancing lesion boundary saliency.	(1) Unstable training and prone to mode collapse. (2) Strongly affected by the balance between generator and discriminator. (3) Limited generalization ability.	Suitable for dermoscopic color and texture anomalies, endoscopic mucosal abnormalities, and relatively obvious local lesions in X-ray images.	High
Image Reconstruction	Diffusion model-based methods	Restore normal structures through progressive denoising and detect anomalies from reconstruction discrepancies.	(1) High generation quality and relatively stable training. (2) Strong capability for recovering complex structures. (3) More suitable for fine-grained localization.	(1) Slow inference and high computational cost. (2) Weak local anomalies may still be over-restored. (3) High deployment cost.	Suitable for complex anomalies such as pulmonary nodules/pneumonia in CT, brain lesions in MRI, blurred-boundary masses in ultrasound, and subtle polyps in endoscopy.	High
Feature Embedding	One-class classification methods	Learn a compact decision boundary of normal samples in feature space.	(1) Simple to implement. (2) Convenient for normal modeling with limited samples. (3) Suitable as an early baseline method.	(1) Difficult to model complex and multi-modal normal distributions. (2) Limited adaptability to medical images with large inter-subject variations. (3) Weak localization ability.	More suitable for anomalies with obvious overall distribution shifts, such as severe thoracic abnormalities in chest X-rays and large lesions in brain MRI.	Low
Feature Embedding	Pre-trained feature distribution modeling methods	Build normal statistical distributions using pre-trained features and detect deviations from them.	(1) Can leverage mature visual representations. (2) Generally provides stable feature representations.	(1) Semantic gap exists between natural and medical images. (2) Limited representation of modality-specific structures. (3) Strongly influenced by the pre-trained backbone.	Suitable for tasks with recognizable visual patterns, such as pulmonary abnormalities in X-ray, lung/kidney structural abnormalities in CT, and vascular/optic disc abnormalities in fundus images.	Moderate
Feature Embedding	Memory-based methods	Store normal prototypes and quantify anomalies through retrieval and matching.	(1) Explicitly preserve normal reference templates. (2) Strong capability for local anomaly localization. (3) Suitable for modeling diverse normal patterns.	(1) Depend on large-scale feature storage and retrieval, leading to relatively high memory and time cost. (2) Poor prototype selection may impair generalization.	Suitable for vascular/optic disc abnormalities in fundus images, endoscopic polyps, local structural disorder in pathology, and local lesions in X-ray images.	Moderately high
Feature Embedding	Normalizing flow-based methods	Model the likelihood distribution of normal features through invertible transformations.	(1) Possess explicit probabilistic modeling capability. (2) Anomaly scores are relatively interpretable. (3) Suitable for multi-scale distribution modeling.	(1) Training and optimization in high-dimensional spaces are relatively complex. (2) Sensitive to conditional modeling design. (3) Relatively high training and inference cost.	Suitable for tasks with evident distribution abnormalities, such as ground-glass nodules in CT, complex lesions in MRI, layered anomalies in OCT, and multi-scale tissue abnormalities in WSI.	High
Feature Embedding	Teacher–student models	The student network mimics normal teacher features, and feature discrepancies are used as anomaly scores.	(1) Feature deviation is intuitive. (2) Usually provides good localization ability with relatively lightweight implementation.	(1) Performance depends on the quality of teacher representations. If teacher features are not well adapted to medical scenarios, performance may be limited. (2) Limited interpretability for complex semantic anomalies.	Suitable for local lesions in chest X-rays, layered structural disorder in OCT, small lesions in MRI, and boundary abnormalities in CT.	Moderate
Self-Supervised Learning	Proxy task-based methods	Learn normal patterns by constructing pseudo anomalies or structural corruption tasks.	(1) Do not require manual annotations. (2) Can improve sensitivity to local structural disruption. (3) Suitable as a representation enhancement mechanism.	(1) Semantic discrepancy may exist between pseudo anomalies and real anomalies. (2) Poor task design may weaken generalization ability.	Suitable for occlusion-like abnormalities in X-ray, local mucosal abnormalities in endoscopy, layered disruption in OCT, and local structural defects in MRI.	Moderate
Self-Supervised Learning	Contrastive learning-based methods	Compress the normal feature distribution through multi-view consistency learning.	(1) Can significantly improve representation discriminability. (2) Helps compact normal feature distributions. (3) Easy to combine with reconstruction or distribution modeling.	(1) Highly dependent on augmentation strategies and positive/negative sample construction. (2) Inappropriate transformations may damage medical semantics.	Suitable for fine-grained vascular abnormalities in fundus images, subtle layer changes in OCT, local semantic shifts in MRI, and texture anomalies in pathology.	Moderate
Foundation Models	Vision–language model-based methods	Perform anomaly discrimination using image–text alignment.	(1) Strong semantic representation ability. (2) Can enhance interpretability through prompt learning. (3) Suitable for open-set anomaly detection.	(1) Depend on prompt design and image–text alignment quality. (2) Limited fine-grained spatial localization ability. (3) Difficult to adapt to medical-specific semantics.	Suitable for text-describable abnormalities such as pneumonia/effusion in chest X-rays, semantic abnormalities in pathology, and cross-category open-set anomalies.	High
Foundation Models	Visual foundation model-based methods	Use large-scale visual pre-trained features for anomaly modeling.	(1) Strong visual representation with relatively good cross-domain generalization. (2) No reliance on textual prompts. (3) Suitable for building unified purely visual anomaly detection frameworks.	(1) Less interpretable than VLM/LLM-based methods. (2) Still insufficient for extremely subtle medical anomalies. (3) Performance depends on the visual foundation model itself.	Suitable for complex visual and multi-scale anomalies across modalities such as CT, MRI, fundus, endoscopy, and WSI.	Moderately high
Foundation Models	Large language model-based methods	Use language reasoning and medical knowledge to enhance anomaly analysis.	(1) Helpful for generating diagnostic explanations and semantic reasoning results. (2) Improves clinical readability. (3) Suitable for complex open scenarios.	(1) Mainly serve high-level semantic analysis, while low-level anomaly detection still depends on visual models. (2) High computational cost. (3) Stability depends on multi-modal collaboration quality.	More suitable for report-level chest X-ray abnormalities, complex case reasoning, and cross-modal medical anomaly analysis.	High

## Data Availability

This study is based on previously published works, and no new data were generated or analyzed. Therefore, data sharing is not applicable.
